# The hormone–microbiota–NET framework in gynecologic diseases: evidence, controversies, and translational opportunities

**DOI:** 10.3389/fimmu.2026.1929292

**Published:** 2026-09-28

**Authors:** Huimin Xu, Tan Jiang, Muxi Li, Deyou Jiang

**Affiliations:** 1 College of Basic Medicine, Heilongjiang University of Chinese Medicine, Harbin, Heilongjiang, China; 2 Department of Chinese Language and Literature, Faculty of Arts, Hong Kong Baptist University, Hong Kong, China; 3 First Clinical Medical College, Heilongjiang University of Chinese Medicine, Harbin, Heilongjiang, China

**Keywords:** gynecologic diseases, hormones, microbiome, neutrophil extracellular traps, pad4

## Abstract

Gynecologic diseases, including infectious disorders, endocrine-related conditions, gynecologic malignancies, and pregnancy complications, are clinically diverse yet frequently share features of hormonal imbalance, microbial dysbiosis, and dysregulated inflammation. Current research has largely examined these processes separately, limiting integrated understanding of their potential interactions. This review synthesizes current evidence and proposes a hormone–microbiota–neutrophil extracellular trap (NET) framework as a conceptual tool for understanding common and disease-specific mechanisms across gynecologic disorders. We first summarize the core molecular basis of NET formation, with emphasis on peptidylarginine deiminase 4 as a candidate regulatory node. We then examine the effects of sex hormones on NET regulation, the role of the microbiota in shaping mucosal immune responses, and potential mechanistic links between microbial products, extracellular matrix components, and NET dysregulation. Evidence is further integrated across representative infectious, endocrine-related, malignant, and pregnancy-associated conditions, including pelvic inflammatory disease, bacterial vaginosis, endometriosis, polycystic ovary syndrome, ovarian, cervical, and endometrial cancers, and preeclampsia. The available literature indicates that hormone-dependent immune modulation, pathogen- or microbiota-associated inflammatory signaling, and NET-related tissue injury may converge in multiple gynecologic settings, although the strength of evidence differs substantially across specific pathways and diseases. Direct support is strongest for selected hormone–NET effects in defined experimental or pregnancy-associated settings and for pathogen/microbial product–NET interactions in infectious contexts, whereas the complete hormone–microbiota–NET framework and the proposed glycosaminoglycan–peptidylarginine deiminase 4 (GAG–PAD4) bridge remain insufficiently validated. Overall, this review provides an evidence-based integrative framework for organizing current knowledge, identifying mechanistic gaps, and informing future biomarker development, patient stratification, and translational research in gynecologic disease.

## 1 Introduction

The spectrum of gynecologic diseases encompasses a wide range of conditions, including infectious disorders, endocrine disturbances, malignancies, and pregnancy-related complications. Population-level analyses from the Global Burden of Disease studies indicate that diseases of the female reproductive system contribute substantially to morbidity and disability worldwide, although estimates vary according to disease definitions, age groups, and analytic categories (Y. 1, 2). Endometriosis, uterine fibroids, pelvic inflammatory disease (PID), and gynecologic malignancies also exert substantial effects on patients’ quality of life (3). Despite their heterogeneous clinical manifestations, these diseases share several common pathogenic features, notably immune dysregulation, endocrine imbalance, and alterations in the microbiota (4, 5).

The immune system plays a central role in gynecologic diseases (6, 7). Pelvic inflammatory disease is characterized by an acute inflammatory response triggered by pathogens, endometriotic lesions exhibit a chronic inflammatory microenvironment, and gynecologic tumors often display immune evasion. Although these three disease settings present distinct immune phenotypes, all involve dysfunctional immune regulation (5, 8–10). The endocrine system is also closely implicated in gynecologic disorders. Endometriosis is estrogen-dependent, polycystic ovary syndrome (PCOS) is characterized by hyperandrogenism, and premature ovarian insufficiency manifests as a decline in endocrine function (11, 12). In recent years, major advances have also been made in research on the reproductive tract microecology. Existing evidence suggests that vaginal dysbiosis is associated with a range of gynecologic conditions, including bacterial vaginosis, persistent human papillomavirus (HPV) infection, pelvic inflammatory disease, and preterm birth. These findings indicate that the microbiota may play an important role in the onset and progression of gynecologic diseases (13–16).

The discovery of neutrophil extracellular traps (NETs) has provided a new perspective for understanding these complex mechanisms. NETs are web-like fibrous structures released by activated neutrophils and consist of a DNA scaffold decorated with histones and granular proteins such as neutrophil elastase (NE) and myeloperoxidase (MPO). They were initially described as an antimicrobial mechanism that contributes to host defense by trapping and killing pathogens (17). However, subsequent studies have shown that excessive NET formation or impaired NET clearance may result in tissue injury, promote fibrosis, and even contribute to tumor progression (18–21).

In the field of gynecology, the involvement of NETs has been reported in multiple settings, although the quality and directness of evidence differ across diseases. NET-related tissue injury has been described in experimental PID/SPID-related settings, NET deposition or NET-associated markers have been reported in endometriosis, NET-related inflammatory changes have been linked to PCOS in exploratory clinical and animal studies, and aberrant NET-related activity has also been implicated in pregnancy complications such as preeclampsia (22–24). Given the broad involvement of NETs in gynecologic diseases, efforts have increasingly turned toward the development of integrative theoretical frameworks. Recently, Wu et al., in a review published in *Frontiers in Medicine*, integrated NETs into gynecologic endocrinology and proposed the conceptual framework of a “NET–sex hormone axis,” suggesting that sex hormones, particularly estrogen and progesterone, regulate NET formation, while NETs may, in turn, influence the progression of hormone-related diseases (5, 25, 26). This framework provides a theoretical basis for understanding the role of NETs in hormone-dependent gynecologic diseases.

The reproductive tract microbiota is a dynamic ecosystem that is profoundly regulated by sex hormones (13, 27). Estrogen promotes glycogen deposition and the growth of *Lactobacillus*, thereby helping maintain an acidic pH and mucosal barrier function, whereas progesterone alters the colonization environment of the microbiota by affecting the properties of cervical mucus (28, 29). The microbiota may regulate local immune responses through multiple mechanisms, including potential effects on neutrophil activation and NET formation (21, 30). Recent *in vitro* studies suggest that microbial metabolites may influence the composition and sulfation patterns of mucosal glycosaminoglycans (GAGs), while GAGs can bind to peptidylarginine deiminase 4 (PAD4), a key enzyme in NETosis, and modulate its calcium sensitivity *in vitro* (31–33). This *in vitro* finding offers a new molecular perspective for understanding the link between the microbiota and NETs. At the same time, NETs themselves may exert feedback effects on the endocrine microenvironment through several possible routes, including protease-mediated degradation of the extracellular matrix, inflammatory factor-mediated effects on the hypothalamic–pituitary–ovarian axis, and fibrosis-associated alterations in local hormone metabolism. However, these pathways are currently supported mainly by correlative studies or *in vitro* experiments, and their causal relationships remain to be clarified (18–20).

Terminologically, “microbiota” refers here to microbial communities, whereas “microbiome” is used only when referring to the broader microbial ecosystem, microbial genetic content, or the wording of cited literature. Because the proposed framework focuses primarily on microbial communities and host immune responses, “microbiota” is used throughout for the mechanistic framework.

Accumulating evidence suggests that inflammatory imbalance in gynecologic diseases is unlikely to be driven by a single factor alone, but may involve interactions among hormonal changes, local microbiota dysbiosis, defined pathogens or microbial products, and aberrant NET formation. From this perspective, examining gynecologic diseases through the lens of a hormone–microbiota–NET framework may help organize shared inflammatory regulatory mechanisms across different disease categories. Current evidence on the hormonal regulation of NETs is derived primarily from *in vitro* studies and animal models outside the gynecologic field, indicating that estrogenic signaling may enhance NETosis under selected conditions, whereas progesterone may restrain selected stages of NET formation; however, direct evidence in gynecologic patients remains relatively limited. Moreover, although pathogen- or microbial product-induced NET formation through pathogen-associated molecular pattern–Toll-like receptor (PAMP–TLR) pathways has been investigated to some extent in acute infection, its role in chronic inflammatory gynecologic disorders remains to be further defined. The proposed framework is illustrated in **Figure 1**.

**Figure 1:**
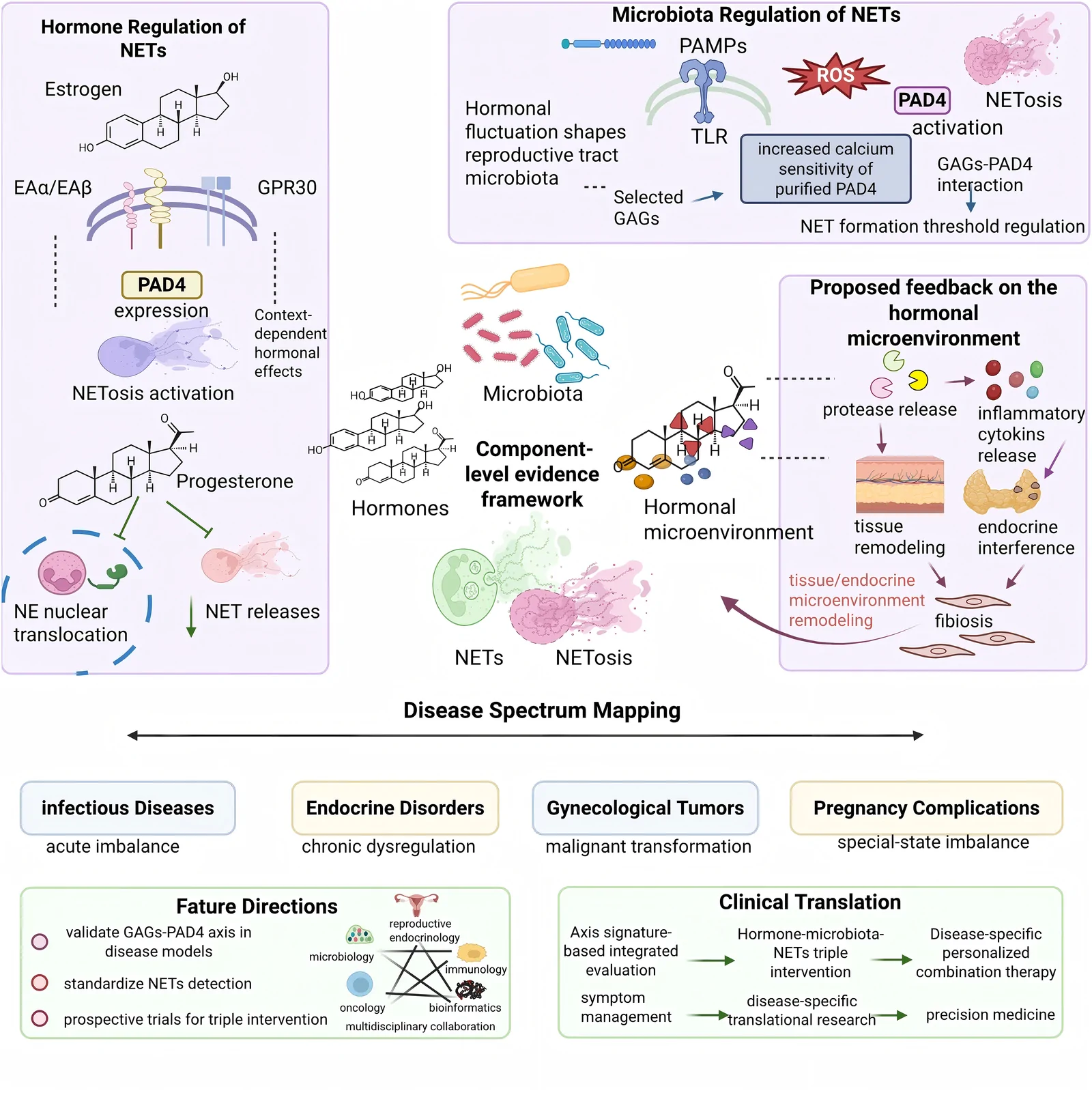
Evidence framework of hormone–microbiota–NET interactions in gynecologic diseases and their translational implications. This figure summarizes the current evidence landscape linking hormonal regulation, reproductive tract microbiota, and neutrophil extracellular traps (NETs) across gynecologic diseases. Rather than representing a unified causal pathway, the framework distinguishes experimentally supported relationships from hypothesis-generating connections. Hormonal effects on NET formation are context-dependent and are supported mainly by experimental systems, pregnancy-associated studies, and non-gynecologic inflammatory models. Microbial stimulation of NET formation through pathogen-associated molecular patterns (PAMPs), Toll-like receptor (TLR) signaling, and reactive oxygen species (ROS) represents a relatively established mechanism in infectious settings. By contrast, the potential regulation of NET formation through microbiota-associated alterations in glycosaminoglycans (GAGs) and PAD4 remains a hypothesis-generating mechanism supported primarily by biochemical studies. The figure further illustrates that NET-associated inflammation, tissue remodeling, and fibrosis may contribute to disease-specific pathological processes; however, the feedback effects of NETs on local hormonal environments remain incompletely validated. Disease categories shown in the lower panel represent contexts in which different components of this framework have been investigated, rather than evidence for a shared universal mechanism. Solid arrows indicate relationships supported in defined experimental or clinical contexts, whereas dashed arrows indicate proposed but unvalidated connections.

### 1.1 Literature identification and evidence assessment

This article was designed as a narrative and hypothesis-generating review rather than a systematic review, with the aim of integrating mechanistic evidence across reproductive endocrinology, reproductive tract microbiology, neutrophil biology, and gynecologic disease. Relevant literature was identified through targeted and iterative searches of PubMed/MEDLINE using combinations of terms including “neutrophil extracellular traps,” “NETosis,” “PAD4” or “PADI4,” “estrogen,” “progesterone,” “microbiome” or “microbiota,” “glycosaminoglycan,” “heparin,” “pathogen-associated molecular patterns,” “Toll-like receptor,” and disease-specific terms corresponding to the conditions discussed in Section 5. Searches were supplemented by backward citation tracking from key primary studies, DOI-linked bibliographic checks, and focused literature updates during revision. Priority was given to peer-reviewed English-language primary studies providing direct human, disease-specific animal, or mechanistic evidence; reviews were used mainly to frame background, terminology, and prior conceptual coverage. Studies of non-gynecologic or non-reproductive systems were retained only when they provided clearly labeled indirect biological plausibility. Non-peer-reviewed material, inaccessible bibliographic records, and studies whose systems did not support the cited claim were not used as claim-defining evidence. Because this was not intended as a systematic review, formal Preferred Reporting Items for Systematic reviews and Meta-Analyses (PRISMA)-based study selection, quantitative meta-analysis, and risk-of-bias assessment were not performed. The literature search covered publications available up to August 2026.

Evidence strength was categorized according to source context and inferential support. Direct evidence denotes human reproductive tract samples, disease-specific gynecologic/reproductive models, or pregnancy-associated systems relevant to the claim. Indirect biological evidence denotes mechanistic support from other tissues, diseases, or purified biochemical systems. Hypothesis denotes an author-proposed connection assembled from separate component findings. When findings conflicted, they were interpreted by sample compartment, assay format, disease context, and experimental system rather than averaged into a single directional claim.

To clarify the conceptual novelty of the present review, **Table 1** summarizes how this article differs from recent NET-, hormone-, microbiome-, cancer-, and PAD4/GAG-focused literature. The table defines the contribution of this review as evidence-mapped integration rather than as a claim that the complete hormone–microbiota–NET framework has already been experimentally validated.

**Table 1:** Conceptual advance of the present review compared with previous reviews and adjacent literature.

Previous literature/representative examples	Main focus or remaining limitation	Conceptual advance of the present review
NETs in gynecologic or female reproductive diseases (5, 34)	Reviews NET involvement and therapeutic opportunities across reproductive diseases; hormone and microbiota contexts are often background.	Adds a component-level map that separates direct gynecologic evidence, indirect extrapolation, and hypotheses.
NET–sex hormone and hormone–NET evidence (26, 35, 36)	Defines possible estrogen- and progesterone-related effects, mainly from pregnancy-associated and experimental systems.	Treats these effects as context-dependent, not as a universal “trigger-and-brake” model.
Reproductive tract and endometrial microbiome reviews (37–39)	Links vaginal/endometrial communities with reproductive health, implantation, pregnancy, cytokines, and growth factors; NET/PAD4 readouts are uncommon.	Connects microbiota to neutrophil/NET biology while separating dysbiosis, defined pathogens, and microbial products.
NET–cancer reviews and ovarian cancer-focused evidence (40–43)	Reviews NETs in metastasis, immune escape, thrombosis, and tumor microenvironment remodeling.	Places gynecologic cancers alongside PID, endometriosis, PCOS, and pregnancy settings using the same evidence categories.
PAD4/GAG and PAD4-inhibition literature (31, 44)	Identifies PAD4 as a target and shows that selected GAGs can increase the calcium sensitivity of purified PAD4.	Defines GAG–PAD4 as a falsifiable compartment-specific hypothesis requiring local GAG/PAD4/NET testing.
Present evidence-mapped synthesis	Evidence is fragmented across endocrinology, microbiology, neutrophil biology, oncology, and pregnancy immunology.	Integrates these fields into one evidence-mapped framework and identifies established, extrapolated, and speculative links.

NETs, neutrophil extracellular traps; PID, pelvic inflammatory disease; PCOS, polycystic ovary syndrome; PAD4, peptidylarginine deiminase 4; GAG, glycosaminoglycan.

## 2 Molecular basis of NET formation

The formation of NETs involves a series of molecular cascades and signaling mechanisms. The following section briefly summarizes the basic pathways of NET formation and the core functions of the key enzyme PAD4, thereby providing the necessary molecular background for discussing the role of the “hormone–microbiota–NET framework” in gynecologic diseases (17, 45–47).

### 2.1 Overview of the three pathways of NET formation

At present, three major regulatory pathways of NETosis are widely recognized: NOX-dependent NETosis (suicidal NETosis), NOX-independent NETosis (vital NETosis), and mitochondrial DNA-derived NETosis (46–48). Although these three pathways differ in their triggering mechanisms and dynamic processes, they ultimately converge on the same endpoint: chromatin decondensation and NET release.

### 2.2 PAD4 as a key enzyme: structure, function, and regulation

PAD4 contributes to histone citrullination and NET formation in selected experimental models, but its involvement should not be generalized to all forms of NET release (49, 50). Its nuclear localization, calcium dependence, and histone citrullination activity belong to relatively well-established general molecular mechanisms, whereas disease-specific regulation of PAD4 in gynecologic disorders still relies largely on extrapolation from other systems (5, 44). Owing to its nuclear localization signal (NLS), PAD4 is able to enter the nucleus and directly act on histones by catalyzing the citrullination of arginine residues in the tails of histones H3 and H4 (51–53). This modification neutralizes the electrostatic interaction between histones and the DNA backbone, thereby promoting chromatin decondensation and creating the conditions required for NET release (44, 49, 54). PAD4 enzymatic activity is strictly dependent on calcium binding (55, 56). Biochemical assays have shown that half-maximal activation of PAD4 requires calcium concentrations of 300–600 μM, in striking contrast to the approximately 100-nM intracellular calcium concentration in resting cells. This discrepancy suggests that additional, as yet unidentified, cofactors may participate in the regulation of PAD4 activity. Moreover, sex-related differences in PAD4 expression have been reported: PAD4 levels are significantly higher in bone marrow-derived neutrophils from women than from men, a finding that may have potential relevance to the direction of “hormone–NET” regulation discussed in this review (57).

### 2.3 The hypothesis-generating GAG–PAD4 mechanism: *in vitro* findings and an *in vivo* hypothesis awaiting validation

#### 2.3.1 Biochemical evidence for GAG-mediated modulation of PAD4

A recent primary biochemical study provided evidence that GAGs can enhance PAD4 activation at sub-saturating calcium concentrations. Bereta et al. demonstrated high-affinity binding between PAD4 and heparin and showed that heparin increased the calcium sensitivity of PAD4 in several purified biochemical assays. In peptide-substrate calcium-response experiments, 0.025 μM heparin lowered the calcium concentration required for half-maximal activation. In a BAEE-based assay, 1 μM heparin reduced the Ca_0.5_ from 370 ± 26 μM to 101 ± 19 μM, and 1 μM heparin also enhanced histone H3 citrullination at lower calcium concentrations. Heparan sulfate, dermatan sulfate, and several chondroitin sulfates showed varying PAD4-activating capacities. The effect depended on GAG chain length and negative charge and required PAD4 dimerization (31).

Importantly, these findings were obtained primarily in purified biochemical systems, and we did not identify an independent primary study directly replicating the GAG-mediated reduction in the calcium requirement of PAD4. These observations therefore establish biochemical plausibility but do not by themselves demonstrate that GAG-dependent PAD4 sensitization occurs in neutrophils or gynecologic mucosal tissues *in vivo*.

#### 2.3.2 Physiological relevance of the experimental GAG concentrations

The physiological relevance of the concentrations and molecular species used in these biochemical assays remains uncertain. Bereta et al. used 0.025 μM heparin in peptide-based calcium-response experiments and 1 μM heparin in BAEE- and histone-based assays, while other natural GAGs were generally evaluated at micromolar concentrations. These experimental conditions cannot currently be assumed to reproduce the concentration, molecular size, sulfation pattern, or bioavailability of free GAG species in the cervicovaginal microenvironment.

Importantly, the study by Myers et al. quantified sulfated GAGs in human cervical tissue rather than free GAG concentrations in vaginal secretions. A recent human study has provided more direct evidence that heparan sulfate is measurable in the vaginal environment: Yano et al. reported higher heparan sulfate concentrations in vaginal swab-derived conditioned medium from women with symptomatic vulvovaginal candidiasis than in asymptomatic and healthy groups. However, because the vaginal swabs were eluted into 1 mL of medium and because heparan sulfate is structurally heterogeneous, these measurements cannot be directly converted into an *in situ* molar concentration comparable with the purified heparin concentrations used in the PAD4 assays (58, 59). Thus, whether GAG concentrations sufficient to sensitize PAD4 occur locally *in vivo* remains unresolved.

#### 2.3.3 Alternative interpretations of the GAG–PAD4 interaction

Several alternative interpretations should be considered before assigning the GAG–PAD4 interaction a causal role in gynecologic NETosis. First, because the core calcium sensitization experiments were performed using purified PAD4, GAG-mediated activation may be particularly relevant to extracellular PAD4 and extracellular protein citrullination rather than necessarily reflecting intracellular PAD4 activation during NETosis. Second, the strong dependence of the effect on polymer length, negative charge, and PAD4 oligomerization raises the possibility that GAGs act as polyanionic allosteric scaffolds rather than through a highly specific disease-related GAG signaling pathway. Third, altered GAG abundance or sulfation may arise downstream of epithelial injury, inflammation, or extracellular matrix remodeling, creating the possibility of reverse causality rather than a unidirectional microbiota → GAG → PAD4 sequence. Finally, GAGs may influence neutrophil function independently of PAD4. For example, elevated vaginal heparan sulfate has been associated with impaired neutrophil–*Candida albicans* interactions and reduced antifungal activity (59).

Accordingly, the microbiota–GAG–PAD4–NET sequence should currently be regarded as one testable mechanistic model among several alternatives rather than as an established causal pathway.

## 3 Regulation of NETs by sex hormones

The regulation of NETs by sex hormones constitutes a key component of the hormone–microbiota–NET framework. Current evidence supports a context-dependent tendency for estrogenic signaling to enhance NET formation and for progesterone to restrain selected stages of NET release, particularly in pregnancy-associated and experimental systems. However, this evidence is still derived primarily from *in vitro* experiments, cell models, and animal studies outside the gynecologic field, while direct validation in gynecologic patient samples and local lesion microenvironments remains limited. Accordingly, this line of evidence is better viewed as providing biological plausibility for the hormonal regulation of NETs rather than as a universally established mechanism across all gynecologic diseases (35, 36).

### 3.1 Estrogen-associated modulation of NET formation: model dependence and receptor evidence

Current evidence that estrogenic signaling can modulate NETosis comes mainly from neutrophil-like cell models, non-gynecologic inflammatory settings, and animal studies and therefore relies largely on cross-system extrapolation, with limited direct evidence from gynecologic contexts. Existing studies suggest that estrogen may enhance the propensity for NETosis in some experimental settings, including membrane estrogen receptor-related pathways, while primary neutrophil responses can vary by donor sex and estrogen concentration; these findings support a context-dependent interpretation rather than a universal estrogen-driven mechanism (26, 36).

In neutrophil-like differentiated HL-60 cells, E2 and the GPR30 agonist G-1 increased NET formation and were accompanied by changes in PAD4 expression and histone citrullination. Pharmaco-logical GPR30 antagonism attenuated these responses (26). These observations support GPR30 involvement in the tested cell model but do not, by themselves, establish a complete receptor-to-PADI4 transcriptional pathway in primary reproductive tract neutrophils. Accordingly, the detailed transcription-factor cascade and a fixed rapid-versus-slow receptor division are not assumed in this review.

The direction of estrogen-associated effects is not uniform across experimental settings. In lipopolysaccharide (LPS)-stimulated primary human neutrophils, higher-than-circulating E2 concentrations suppressed NET formation in male-derived cells, whereas concentrations near the upper non-pregnant range tended to increase NET formation in female-derived cells (36). These findings favor a context-dependent interpretation rather than a universally pro-NETotic effect of estrogen.

### 3.2 Progesterone-associated restraint of NET release in pregnancy-related experiments

In pregnancy-associated experiments, progesterone reduced NET release under the conditions examined, while histone citrullination persisted and NE nuclear translocation was restricted (35). These findings identify a context-specific regulatory effect but do not establish a universal block of late-stage NET formation. The receptor-proximal events linking progesterone exposure to altered NE trafficking remain insufficiently resolved in the evidence reviewed here. Extension to non-pregnant gynecologic lesions, or a failure of this regulatory effect in preeclampsia, requires disease-specific validation.

### 3.3 Context dependence of combined hormonal effects

The opposing effects of estrogen and progesterone have been reported in selected pregnancy-associated and experimental settings, but the evidence reviewed does not establish a universal “trigger-and-brake” system across gynecologic diseases. **Figure 2** therefore distinguishes model-specific observations from untested receptor-level mechanisms and disease-level extrapolations. Whether comparable interactions occur within the same non-pregnant lesion remains an open research question.

**Figure 2:**
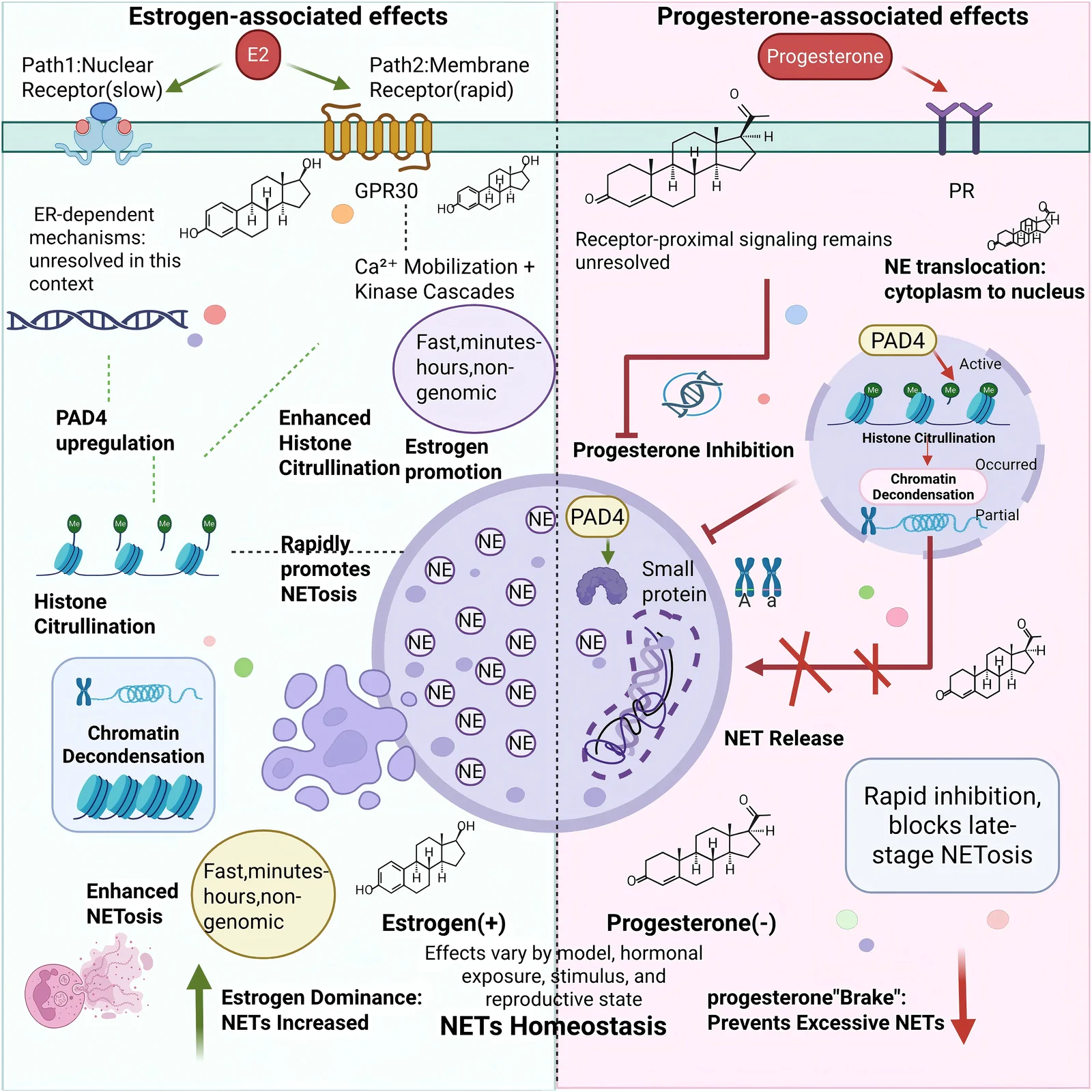
Context-dependent hormonal modulation of neutrophil extracellular trap (NET) formation. This figure illustrates experimental evidence suggesting that estrogen- and progesterone-associated signaling can influence NET formation under specific biological conditions. Current evidence does not establish a universal “estrogen trigger–progesterone brake” system across gynecologic diseases. Estrogen-associated enhancement of NET-related responses has been reported in selected neutrophil models, including studies involving estrogen receptor signaling and membrane receptormediated pathways. These findings suggest possible regulation of PAD4 expression, histone citrullination, or NET-associated responses; however, the responsible molecular intermediates and their relevance in gynecologic tissues remain incompletely defined. Progesterone-associated inhibition of NET release has mainly been described in pregnancy-related immune settings, where progesterone may influence later stages of NET formation, including neutrophil elastase (NE) nuclear translocation. The downstream mechanisms linking progesterone receptors to cytoskeletal regulation and vesicular processes remain unresolved. Therefore, this figure represents model-specific hormonal modulation rather than a general physiological rule. Solid arrows indicate experimentally observed effects under defined conditions, whereas dashed arrows indicate proposed mechanisms requiring further validation.

### 3.4 Potential menstrual-cycle variation in NET formation

The hormonal effects discussed in Sections 3.1–3.3 raise the possibility that NET formation varies across the menstrual cycle. However, the evidence reviewed here does not establish a reproducible cycle-dependent NET pattern in the non-pregnant reproductive tract, and findings from experimental or pregnancy-associated settings should not be directly extrapolated to menstrual physiology. This possibility should therefore be regarded as a testable hypothesis rather than an established physiological model. Longitudinal studies combining cycle-phase assessment, hormone measurements, and standardized NET evaluation are needed to determine whether such variation occurs (36, 60).

### 3.5 Molecular basis of sex differences

Women show greater susceptibility than men to a variety of autoimmune diseases and NET-related disorders, a phenomenon long thought to be associated with sex hormones. In recent years, mouse studies have shown that Padi4 expression is higher in bone marrow-derived neutrophils from women than from men and that this difference displays a certain degree of cell type specificity. Following intervention with PAD4 inhibitors, female animals exhibited a more pronounced reduction in NETs and greater functional improvement. These findings provide a possible molecular explanation for the greater tendency of women to develop NET-associated pathological changes (57, 61).

## 4 The bridging role of the microbiota: constructing a three-component interaction network

The reproductive tract microbiota represents a key link connecting hormonal signaling with NET-mediated effects and is also the most integrative yet most unevenly supported component of the hormone–microbiota–NET framework. Overall, hormone-associated changes in microbiota composition are supported by relatively direct human observational evidence in gynecology, and defined pathogens or microbial products can induce NETs through pattern recognition pathways in infectious settings. By contrast, the idea that community-level microbiota alterations may change the threshold for NET formation through a GAG–PAD4 route, and that NETs may, in turn, remodel the hormonal microenvironment, remains supported mainly by *in vitro* biochemical evidence and hypothesis-based reasoning (**Figure 3**).

**Figure 3:**
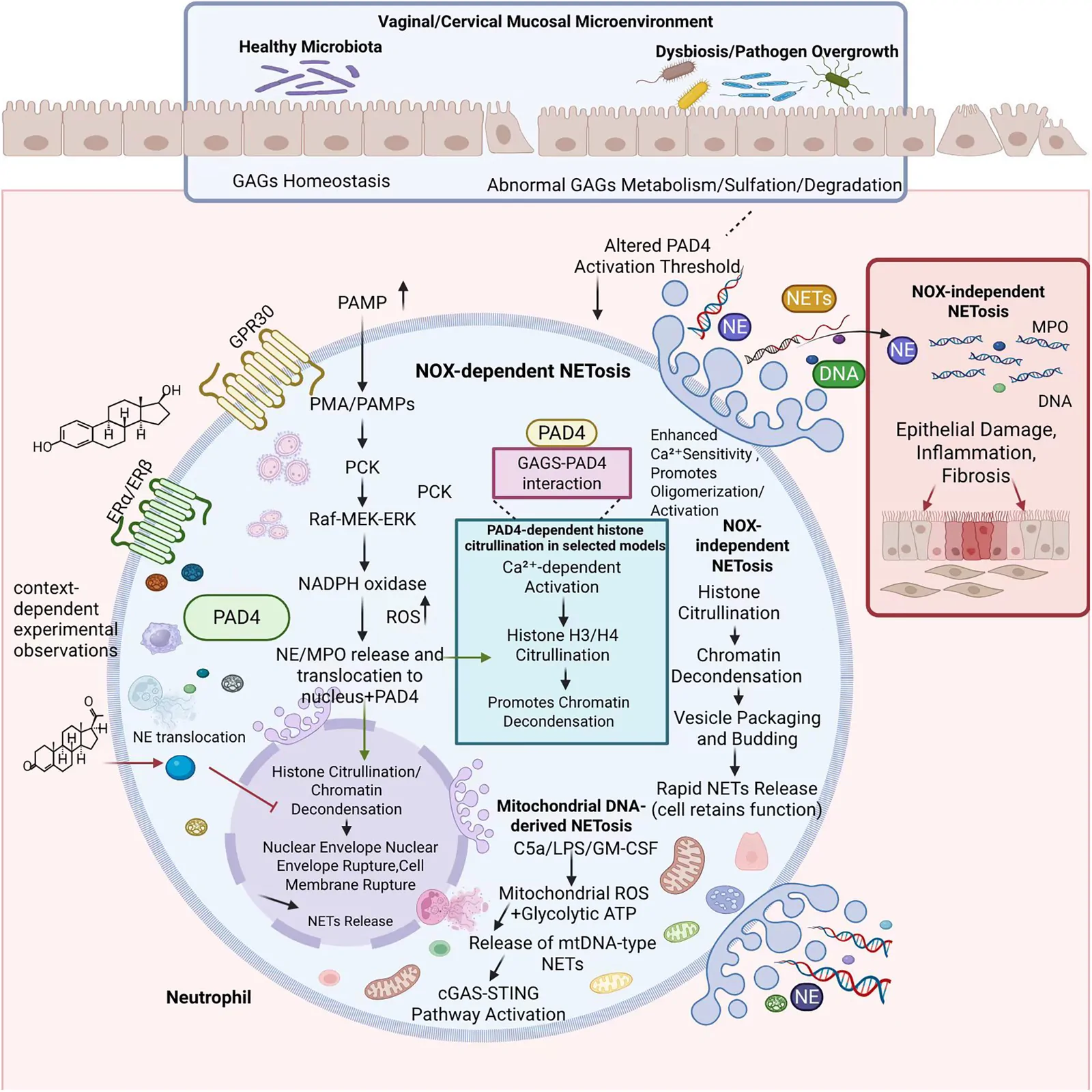
Hypothesis-generating model of GAG-related regulation of PAD4 and NET formation in the reproductive tract microenvironment. This figure presents a hypothesis-generating model describing potential relationships among microbial changes, local glycosaminoglycan (GAG) environments, peptidylarginine deiminase 4 (PAD4) regulation, and neutrophil extracellular trap (NET) formation in the reproductive tract. Direct pathogen-associated stimulation of neutrophils through inflammatory pathways such as pathogen-associated molecular pattern–Toll-like receptor–reactive oxygen species (PAMP–TLR–ROS) signaling represents the better-supported mechanism for NET induction in infectious contexts. By contrast, the proposed relationship between altered local GAG composition, PAD4 activation sensitivity, and NET formation is based primarily on purified biochemical studies showing that selected GAGs can enhance PAD4 calcium sensitivity. Whether microbiota-associated GAG alterations occur in gynecologic tissues, whether these changes influence intracellular PAD4 activity in neutrophils, and whether they modify NET formation in vivo remain unresolved questions. The GAG–PAD4 interaction shown here should therefore be interpreted as a testable mechanistic hypothesis rather than an established disease pathway. Solid arrows indicate experimentally demonstrated effects within defined systems, whereas dashed arrows indicate proposed biological links requiring validation.

### 4.1 Hormonal regulation of the reproductive tract microbiota (first link)

The relationship whereby hormones shape the composition of the reproductive tract microbiota is supported by relatively direct human observational evidence in gynecology, although marked heterogeneity remains across anatomical sites, hormonal states, and populations (62–66). Current studies suggest that estrogen plays a key role in maintaining a healthy vaginal microbiota. First, estrogen promotes proliferation of vaginal epithelial cells and glycogen deposition. Glycogen is then metabolized by *Lactobacillus* into lactic acid, thereby maintaining an acidic environment and suppressing pathogen growth. Second, human studies suggest that 17-β estradiol levels are associated with vaginal microbiota structure, with higher estrogen levels generally linked to the more typical microbiota composition seen during the reproductive years. Third, the postmenopausal decline in estrogen is often accompanied by reduced *Lactobacillus* abundance, increased microbial diversity, and elevated vaginal pH, providing relatively direct human evidence that estrogen contributes to the maintenance of *Lactobacillus* dominance.

However, the hormone–microbiota relationship is not simply linear. Some studies have shown that, in specific settings such as *in vitro* fertilization (IVF), supraphysiological estrogen levels do not significantly alter the abundance of certain microbial populations within the endometrial cavity, suggesting that hormonal effects may be subject to threshold effects, compartment specificity, and interindividual variation. Compared with estrogen, progesterone has been studied less extensively in relation to the microbiota; available evidence more cautiously supports hormone- and treatment-associated variation in cervicovaginal or endometrial environments rather than a single progesterone-driven pattern (28, 62, 67).

Accordingly, hormonal shaping of the reproductive tract microbiota represents one of the more directly supported components of this framework. Even so, this relationship is influenced by multiple factors, including anatomical site, age, menopausal status, pregnancy status, and treatment background, and should not yet be generalized into a single fixed pattern (68).

Endometrial and vaginal microbial findings should also be interpreted according to anatomical compartment. A recent maternal–fetal interface review emphasized that the endometrial microbiome may act together with cytokines and growth factors during implantation and early pregnancy, including through modulation of maternal immune adaptation and the local embryo-supportive environment (39). In the present framework, this literature supports a microbiota–immune microenvironment connection but does not provide direct evidence for microbiota-driven PAD4 activation or NET formation.

### 4.2 Microbiota-, pathogen-, and microbial product-mediated regulation of NETs (second link)

The relationship between microbial communities, defined pathogens, microbial products, and NETs is a key but unevenly supported component of the hormone–microbiota–NET framework. Three related but non-interchangeable propositions should be distinguished. First, defined pathogens or microbial products can trigger neutrophil activation and NETosis through PAMP–pattern recognition receptor–reactive oxygen species (ROS) pathways in infectious settings. Second, community-level dysbiosis may alter the mucosal inflammatory environment and thereby associate with ET/NET abundance. Third, a still-unverified hypothesis proposes that local GAG profiles may be altered and PAD4 activity thereby affected, ultimately modulating the threshold for NETosis (69, 70) (**Figure 4**).

**Figure 4:**
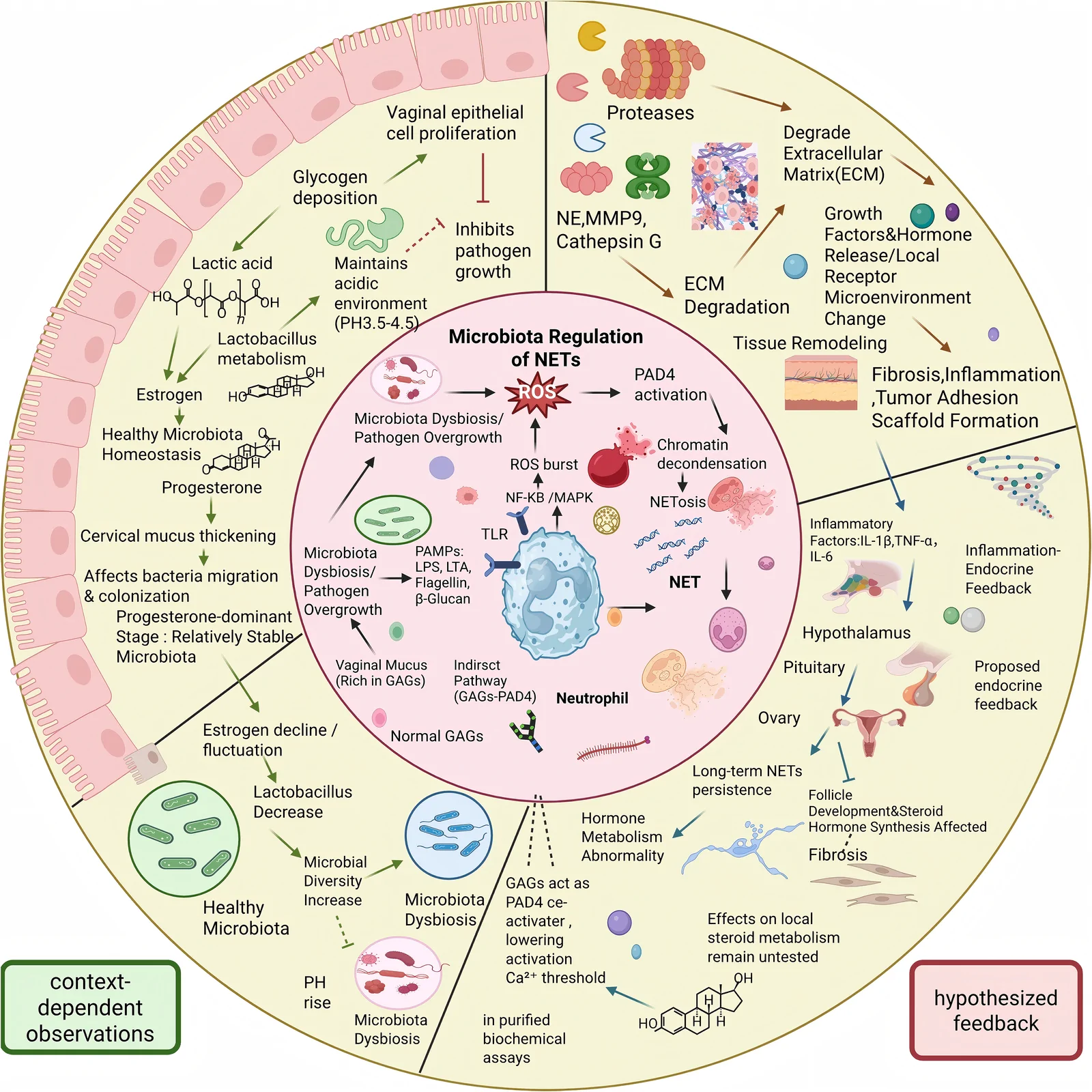
Proposed interactions among microbiota, NET-related inflammation, and mucosal homeostasis imbalance. This figure illustrates potential interactions between reproductive tract microbiota, neutrophil activation, neutrophil extracellular trap (NET) formation, and tissue inflammatory responses. Under physiological conditions, hormone-associated microbial homeostasis may contribute to maintenance of mucosal barrier function. In dysbiotic or infectious conditions, microbial components and inflammatory signals can activate neutrophils and promote NET formation. NETassociated molecules, including proteases and extracellular DNA structures, may contribute to tissue remodeling and inflammatory amplification. However, the directionality and causal relationships among microbiota alterations, NET formation, tissue injury, and local endocrine changes remain incompletely established. In particular, the proposed glycosaminoglycan (GAG)-related modulation of peptidylarginine deiminase 4 (PAD4) activity and NET-mediated feedback effects on hormonal environments require further experimental validation. This figure therefore represents an integrative hypothesis framework rather than a confirmed pathological feedback loop. Solid arrows indicate relatively supported biological relationships, whereas dashed arrows indicate proposed interactions.

#### 4.2.1 Direct pathway: the PAMP–TLR–ROS route

The direct pathway is the classical mechanism that has been studied relatively extensively and is supported by comparatively direct evidence in vaginitis and infection-related inflammatory settings, although its explanatory power for chronic non-infectious gynecologic diseases remains limited (69–71). During pathogen overgrowth or dysbiotic inflammation, microbial products can activate neutrophils through pattern recognition receptor pathways, including TLR-related responses to bacterial products and CR3-associated responses to β-glucan, thereby engaging ROS-dependent or related signaling modules and promoting chromatin decondensation and NET release (69, 72, 73).

A separate but related line of evidence concerns defined microbial products rather than community-level microbiota. In a single-cell RNA-sequencing study, LPS exposure at the maternal–fetal interface altered cellular composition and cell–cell communication, upregulated pro-inflammatory cytokines (including CCL2, TNF-α, and IL-1β), and increased immune-cell populations (74). This study strengthens the relevance of microbial product-driven inflammatory remodeling in reproductive tissues, but it should not be interpreted as proof of community-level dysbiosis-induced NETosis or of a microbiota–GAG–PAD4 pathway.

Studies of vaginitis have provided relatively direct support for this pathway. Increased numbers of NETotic cells have been observed in samples from patients with *Candida* infection and *Trichomonas* infection, and some cases of bacterial vaginosis, suggesting a clear link between pathogen stimulation and NET activation. Animal models of vulvovaginal candidiasis further show that impaired NET formation increases host susceptibility to infection, supporting a defensive role for NETs in infection control (70). However, this evidence is derived mainly from acute infectious settings. Therefore, although the PAMP–pattern recognition receptor–ROS route represents a classical mechanism by which pathogens can directly induce NETs, it should not be automatically extrapolated to NET abnormalities in chronic non-infectious gynecologic diseases such as endometriosis, PCOS, or preeclampsia (70, 71).

#### 4.2.2 A hypothesis-generating GAG–PAD4 mechanism

As summarized in Section 2.3, selected GAGs enhance the calcium sensitivity of purified PAD4 in biochemical assays (31). Extending this observation to gynecologic NET formation requires evidence that microbial changes alter the relevant local GAG species, that these species influence PAD4 in the relevant cellular compartment, and that this interaction changes NET formation. These connections have not been established by the studies reviewed here. The proposed sequence is therefore one testable mechanism among alternatives, including extracellular PAD4 activity, injury-related GAG changes, and PAD4-independent effects on neutrophils.

#### 4.2.3 Potential synergy between the two pathways

These pathways may not operate independently. Defined pathogens or microbial products may rapidly increase NETosis during acute infection, whereas community-level mucosal changes and the GAG–PAD4 mechanism, if validated, may influence baseline sensitivity under chronic conditions by altering the threshold for NETosis. In certain disease states, immediate activation driven by PAMP–pattern recognition receptor–ROS pathways and putative threshold modulation mediated by GAGs–PAD4 may together determine the intensity and duration of NET release (73).

### 4.3 Feedback effects of NETs on the hormonal microenvironment (third link)

Compared with the evidence that hormones influence the microbiota and that the microbiota regulates NETs, the evidence that NETs, in turn, reshape the hormonal microenvironment is currently the most indirect, but also the most prone to overinterpretation. Existing studies suggest that NETs are not merely terminal products of inflammation. Rather, the proteases they carry, their DNA scaffold, and the inflammatory mediators released in parallel may theoretically influence hormonal exposure and endocrine signaling through tissue remodeling, extracellular matrix degradation, propagation of inflammation, and alterations in the local metabolic milieu. Based on current evidence, this potential feedback can be discussed mainly at three levels: protease-mediated tissue remodeling, interference of inflammatory mediators with the hypothalamic–pituitary–ovarian (HPO) axis, and the possible link between NET-associated fibrosis and altered local hormone metabolism.

#### 4.3.1 Protease-mediated tissue remodeling and changes in the hormonal microenvironment

NETs can carry and release highly active proteases such as NE, MMP9, and cathepsin G, all of which may degrade the extracellular matrix and promote tissue remodeling. In tumor and inflammatory settings, extracellular matrix degradation not only alters tissue architecture but may also influence the local signaling microenvironment of matrix-bound growth factors, hormones, and their receptors. In principle, protease activity associated with NETs may therefore indirectly alter local hormonal exposure (75–78).

#### 4.3.2 Potential effects of inflammatory mediators on the hypothalamic–pituitary–ovarian axis

NET formation is accompanied by the release of inflammatory mediators such as IL-1β, TNF-α, and IL-6. Theoretically, these molecules may affect the HPO axis through the circulation, thereby interfering with GnRH pulsatility, gonadotropin secretion, and ovarian steroidogenesis (79). In PCOS and endometriosis, chronic NET-associated inflammation has been correlated with hyperandrogenism, ovulatory dysfunction, or the progression of estrogen-dependent lesions, providing indirect support for a possible NET–inflammation–endocrine connection.

#### 4.3.3 Possible association between NET-related fibrosis and local hormone metabolism

Persistent NETs may promote fibrosis in selected experimental pelvic or inflammatory settings, and changes in hormone metabolism-related enzymes, such as aromatase and 17β-HSD, could theoretically accompany fibrotic tissue remodeling. In endometriosis, NET-related markers and estrogen dependence co-occur, but a direct NET–fibrosis–aromatase/17β-HSD causal pathway has not been demonstrated (8, 12, 80, 81) (**Tables 2**, **3**).

**Table 2:** Component-level evidence map of hormone–microbiota–NET relationships: study systems, supported findings, and untested links.

Component/proposed link	Study system and sampled compartment	Evidence provenance and design	Observed or experimentally supported finding	Unproven link/limit of extrapolation	Representative primary studies
Estradiol → NET formation	Neutrophil-like HL-60 cells; isolated human blood neutrophils exposed to LPS and estradiol.	I: cell-line and primary-cell experiments; hormone exposure and receptor-related perturbation.	E2/G-1 increased NET-related responses in the HL-60 model. In primary neutrophils, estradiol responses varied with donor sex and concentration.	Does not establish a universal estrogen “trigger,” a menstrual-cycle trajectory, or the same response in gynecologic lesions.	Yasuda et al. (26) and Ishikawa et al. (36)
Progesterone → selected stages of NET release	Maternal circulating neutrophils/plasma and *in vitro* hormone experiments in a pregnancy-related study; not local lesion sampling.	D-systemic with *ex vivo*/*in vitro* experiments: pregnancy-associated observations and functional perturbation.	Progesterone restrained NET release and NE nuclear translocation under the tested conditions despite neutrophil priming.	Does not demonstrate the same mechanism in non-pregnant lesions, or failure of this restraint in preeclampsia.	Giaglis et al. (35)
Hormonal context ↔ reproductive tract microbiota	Human vaginal samples across menopausal/cycle contexts; vaginal and endometrial samples during IVF treatment.	D-local: observational comparisons, longitudinal sampling, and a treatment-associated pilot study.	Microbiota composition and/or the local glycogen environment varied with reproductive or treatment context.	These designs do not isolate a single hormone as the cause of every community change. They do not establish downstream NET effects or interchangeability of vaginal and endometrial findings.	Mirmonsef et al. (29)Song et al. (65), and Carosso et al. (62)
Defined pathogens → neutrophil responses/NETs	Human neutrophils challenged with *Trichomonas vaginalis*; human vaginal discharges; mouse vulvovaginal candidiasis.	I: isolated-cell pathogen challenge. D-local: patient observation. D-model: genital infection experiments.	*T. vaginalis* induced NET formation *in vitro*. NET structures were detected in vaginal infection samples; NET-forming capacity was associated with antifungal defense in the mouse model.	Pathogen-specific results do not establish a community-dysbiosis mechanism or a universal TLR–ROS–PAD4 sequence. Detection in a vaginal sample does not itself identify every upstream signaling step.	Ramírez-Ledesma et al. (69)Zambrano et al. (71), and Yano and Fidel (70)
Defined microbial product → uterine NET-related inflammation	LPS-induced mouse endometritis; uterine tissue and inflammatory/NET-related measurements.	D-model: microbial-product challenge with DNase I or Cl-amidine intervention in separate studies.	The interventions attenuated uterine injury/inflammation and NET-related readouts in the tested LPS models.	LPS challenge is not a community-composition experiment or a live polymicrobial infection. It does not establish dysbiosis as the cause or validate the GAG–PAD4 hypothesis.	Hao et al. (82) and Shen et al. (83)
Community-level vaginal dysbiosis ↔ ET/NET abundance	Human vaginal samples classified by BV status; extracellular DNA/LL-37 detection and later cell source-oriented imaging.	D-local: cross-sectional/*ex vivo* structural observations.	ETs were reported in BV samples. A later imaging study distinguished NETs from macrophage-associated traps and reported greater NET abundance in BV.	LL-37-associated DNA alone does not establish neutrophil origin. Co-occurrence does not establish whether dysbiosis drives NETs, NETs alter communities, or both reflect inflammation.	Ramírez-Ledesma et al. (84) and Prado-Sanhueza et al. (85)
BV-associated bacteria → neutrophil recruitment/barrier injury	Experimental female genital tract exposure to selected BV-associated bacterial species; vaginal inflammation and barrier readouts.	D-model: bacterial-exposure experiments with neutrophil/barrier assessment.	Selected bacteria induced neutrophil-driven inflammation, junctional disruption, and increased epithelial permeability.	Neutrophil involvement is not equivalent to NET-specific mediation. These results do not by themselves demonstrate that NETs cause the barrier injury.	Costa-Fujishima et al. (86)
Vaginal microbial context ↔ local glycans/GAGs	Primary vaginal epithelial cells in BV research; vaginal swab-conditioned medium from women with vulvovaginal candidiasis.	D-local: epithelial glycan profiling and sialidase experiments; separate clinical/*ex vivo* heparan sulfate observations.	*Gardnerella* sialidases altered epithelial sialoglycans. Separately, higher vaginal heparan sulfate was linked to impaired neutrophil–*Candida* killing.	Sialoglycan remodeling is not proof of altered GAG sulfation. Neither study establishes intracellular PAD4 sensitization or the complete microbiota–GAG–NET sequence.	Agarwal et al. (87) and Yano et al. (59)
Defined GAGs → PAD4 calcium sensitivity	Purified PAD4 with heparin and other defined GAGs; binding, calcium-response, and substrate-citrullination assays.	I: purified biochemical experiments.	Selected GAGs increased PAD4 activity at sub-saturating calcium; effects differed with GAG species and molecular properties.	Does not establish effective local concentrations, access to intracellular PAD4, or NET formation in reproductive tissues. Heparin and heparan sulfate are not interchangeable.	Bereta et al. (31)
Dysbiosis → altered GAGs → intracellular PAD4 → NETs	Author-proposed sequence linking observations from different systems; no end-to-end reproductive experiment in the mapped studies.	H: integrated mechanistic hypothesis, not an observed causal chain.	The studies above support selected, separate steps only; the complete sequence is proposed for testing.	Requires linked evidence on microbial changes, specific local GAG alterations, PAD4 activity, and NET formation, with relevant perturbation and controls.	Author synthesis: component studies detailed above do not provide evidence for the complete chain.
NET-related processes → tissue remodeling/fibrosis	SPID rat pelvic tissues and dHL-60-derived NET preparations applied to rat uterine fibroblasts; separately, experimental peritoneal adhesions and human adhesion biopsies.	D-model: SPID tissue/cell experiments. I for PID-specific causation: generic peritoneal adhesion studies.	The SPID study linked NET-related/autophagy changes with fibrosis and reported fibroblast responses to NET preparations. DNase-related interventions reduced experimental peritoneal adhesions in a separate study.	Generic peritoneal adhesions are not equivalent to human PID sequelae. Results do not establish a shared hormone–microbiota mechanism; cell preparations and intervention specificity constrain attribution.	Dang et al. (81) and Elrod et al. (88)
NETs → feedback on the hormonal microenvironment	NET-dependent matrix remodeling in mouse lung inflammation/tumor-dormancy models; endocrine feedback proposed by this review.	I: cross-tissue experimental plausibility for matrix remodeling. H: NET-specific endocrine feedback.	NET-associated proteolysis altered the extracellular matrix and supported tumor-cell awakening in the tested lung setting.	The cited experiment does not demonstrate changes in reproductive steroidogenesis, aromatase/17β-HSD activity, or HPO axis regulation. These extensions remain hypotheses.	Albrengues et al. (75); endocrine extension: author hypothesis.

Evidence provenance: D = direct reproductive/gynecologic context, qualified as local samples, disease/pregnancy-associated systemic samples, or disease-specific models; I = indirect biological evidence from other systems; H = an author-proposed hypothesis. These labels identify source context, not study quality or causal certainty. D-systemic does not establish a local tissue mechanism. Arrows indicate the relationship under examination; they do not imply that causality has been established. The “supported finding” and “unproven link” columns delimit each claim. Findings from different pathogens, anatomical compartments, or experimental systems must not be assembled into a validated causal chain. A defined pathogen/product challenge, community dysbiosis, epithelial sialoglycan remodeling, and GAG-dependent PAD4 sensitization are distinct propositions. “Not established” refers to the studies mapped here, not proof that no relevant study exists. No listed evidence establishes the complete hormone–microbiota–NET framework as a shared causal mechanism across diseases.

NET, neutrophil extracellular trap; LPS, lipopolysaccharide; IVF, *in vitro* fertilization; TLR, Toll-like receptor; ROS, reactive oxygen species; PAD4, peptidylarginine deiminase 4; GAG, glycosaminoglycan.

**Table 3:** Disease-specific evidence for hormonal, microbial, and NET-related components: demonstrated connections, anatomical boundaries, and remaining gaps.

Disease/setting	Hormonal component	Microbial component and anatomical site	NET evidence: system, readout, and study	Evidence type/connection actually supported	Missing bridge and cross-system limit	Translational implication
Pelvic inflammatory disease/SPID	Hormonal modulation of NET-related injury was not directly tested in the cited SPID study.	An infection-associated disease context; the SPID model is not evidence that community-level dysbiosis drives the NET response.	Rat pelvic/adhesion tissues; dHL-60 NET preparations and rat uterine fibroblasts. Fibrosis-associated changes and fibroblast proliferation/migration were reported (81).	D-model: tissue observations plus cell experiments; autophagy perturbation supports model-level biology, not a complete triadic mechanism.	Elrod et al. (88) concerns generic peritoneal adhesions: indirect for PID-specific causation. Human hormone–microbiota–NET causality remains unproven.	Study sequelae mechanisms; these experiments do not establish NET-guided treatment in patients.
Bacterial vaginosis	Hormonal context may modify the vaginal environment, but hormonal causation of the observed NET differences was not established.	Vaginal community dysbiosis; distinguish BV classification from detection of a single species such as *Gardnerella vaginalis*.	Human vaginal ET/NET imaging (84, 85). Separate bacterial-exposure models assessed neutrophil-dependent barrier injury (86).	D-local: dysbiosis–ET/NET association. D-model: bacterial effects on neutrophils/barrier. These are related but distinct findings.	ET cell origin is unresolved in the LL-37 study. A NET-specific cause of barrier injury, hormonal mediation, and a GAG–PAD4 connection were not jointly established.	Study local timing and phenotype; clarify antimicrobial defense versus injury before considering NET suppression.
Endometriosis	Estrogen-dependent disease context; direct hormonal control of lesion NETs is not established by the listed NET studies.	Microbiota-related mechanisms are discussed separately in the review; they are not demonstrated by the listed NET observations.	Peritoneal-fluid NET observations (8); plasma NET-associated signal in deep infiltrating disease (80); circulating cf-DNA, nucleosome, and NE measurements (24).	D-local and D-systemic: observational evidence for local/peripheral NET-related abnormalities; not intervention-based proof of lesion causation.	Peripheral markers do not locate NET release to lesions. Direct evidence for NET-driven fibrosis, hormonal mediation, and microbiota–GAG–PAD4 coupling remains incomplete.	Explore local and systemic markers; validate lesion, pain, and infertility mechanisms separately.
Polycystic ovary syndrome	Hyperandrogenic/endocrine-metabolic context; a direct androgen → NET effect was not isolated in the cited disease experiments.	Gut—not vaginal—microbiota transfer/*Bacteroides vulgatus* experiments affected metabolic and ovarian phenotypes through a bile acid/IL-22 pathway (89).	Patient serum/follicular fluid markers; DHEAS-induced PCOS rats with DNase I treatment; hepatocyte responses to NET-conditioned medium (90).	D-local/systemic observations and D-model intervention: a NET-related metabolic branch. Qi et al. supported a separate gut microbiota branch, not demonstrating NET mediation.	The branches must not be joined as a validated microbiota → NET → insulin-resistance sequence. High-fat-diet and soluble-NE studies are indirect, not PCOS-specific NET proof.	Test metabolic and compartment-specific links; neither a genital microbiota–NET mechanism nor human benefit is established.
Ovarian cancer	Hormonal regulation of omental NETs was not established in the cited metastasis model.	The cited NET metastasis study did not demonstrate an upstream reproductive tract microbiota mechanism.	Orthotopic ovarian cancer/omental models: neutrophil recruitment, NET-mediated tumor-cell capture, and reduced omental colonization after neutrophil-specific PAD4 deficiency or pharmacological inhibition (43).	D-model: disease-specific genetic/pharmacological perturbation supports PAD4/NET involvement in omental colonization under the tested conditions.	No evidence for the complete triad or a human treatment effect. General cancer reviews and non-ovarian immune-shielding studies are not substitutes for disease-specific experiments.	Study metastatic niches preclinically; clinical benefit and hormonal or microbial mediation remain unproven.
Cervical cancer	Hormonal mediation of NET induction was not established in the cited tumor studies.	HPV-related tumor signaling is a pathogen-associated context, not proof of community-level bacterial dysbiosis.	Human tumors and experimental models: S100A7-associated NET formation/lymphatic dissemination (91); HPV-associated CXCL8 signaling, tissue/serum NET signals, and tumor-promoting effects (92).	D-local/systemic observations plus D-model/cell perturbation. Experimental NET degradation reduced selected tumor-promoting outcomes; NLR is not the NET readout.	These studies do not establish a hormonal or vaginal community mediator. Findings should not be reduced to NLR associations or generalized across all gynecologic cancers.	Investigate mechanisms and biomarkers; independently validate disease-specific findings before clinical use.
Endometrial cancer	An endocrine-related tumor context does not itself demonstrate an estrogen → PAD4 → NET causal pathway.	A microbial or community-dysbiosis driver of NETs was not established by the listed studies.	Tumor DNA/NE/CitH3 colocalization and circulating measurements (93); blood histone–DNA, cf-dsDNA, and NE, with prognostic associations for cf-dsDNA (94).	D-local: tissue structural observations. D-systemic: clinical biomarker/prognostic associations; not NET-specific causal intervention.	cf-dsDNA is not specific to NETs; its prognostic association does not prove NET-mediated progression. Hormonal and microbial bridges remain untested.	Validate candidate assays and added clinical value; no established NET-targeted treatment follows.
Preeclampsia	Pregnancy-associated hormonal context; failure of the progesterone restraint mechanism was not directly shown.	A reproductive tract community dysbiosis → NET mechanism was not demonstrated in the cited PE studies.	Patient plasma and trophoblast experiments (95); predelivery maternal blood biomarkers (96). Sundet reported lower CitH3 in early/late PE and lower MPO–DNA in late PE.	D-systemic: non-uniform circulating-marker findings. Reproductive-cell experiments support NET-associated inflammatory injury in the tested system, not disease initiation.	Circulating markers are not interchangeable with placental NET deposition. Conflicting directions must be retained; neither hormonal failure nor microbial causation is established.	Validate by compartment, timing, and assay; no uniform “elevated NET” diagnostic pattern is established.

Representative studies are organized by disease. Evidence for an individual component does not imply validation of the complete framework. D, I, and H follow the provenance definitions in **Table 2**. Hormonal or microbial disease context is distinguished from a demonstrated causal link to NETs. Tissue structures, soluble NET-related components, cell-line responses, and circulating inflammatory ratios are not interchangeable evidence. DNase or pharmacological responses support only the outcomes and mechanisms justified by the controls in the relevant study; intervention alone does not validate the complete framework. The studies are representative of this narrative review, not an exhaustive evidence-grading exercise. Translational implications are research directions, not treatment recommendations.

BV, bacterial vaginosis; cf-DNA, cell-free DNA; CitH3, citrullinated histone H3; DHEAS, dehydroepiandrosterone sulfate; E2, estradiol; ETs, extracellular traps; GAGs, glycosaminoglycans; HPO, hypothalamic–pituitary–ovarian; HPV, human papillomavirus; IVF, *in vitro* fertilization; LPS, lipopolysaccharide; MPO, myeloperoxidase; NE, neutrophil elastase; NETs, neutrophil extracellular traps; NLR, neutrophil-to-lymphocyte ratio; PAD4, peptidylarginine deiminase 4; PE, preeclampsia; SPID, sequelae of pelvic inflammatory disease.

## 5 Framework component dysregulation across the spectrum of gynecologic diseases

As an integrative framework, the value of the hormone–microbiota–NET framework does not lie in forcing all gynecologic diseases into a single mechanistic etiologic model, but rather in helping to identify which dysregulated components may predominate in different diseases and how these components may interact. Current evidence suggests that NET-associated abnormalities do occur across multiple categories of gynecologic disease, although the sources of evidence, mechanistic depth, and degree of causal support vary across disease types. In infectious diseases, the evidence that defined pathogens or microbial products can directly induce NETs is relatively strong. In endocrine-related disorders, NETs appear more often as participants in chronic inflammation and tissue remodeling. In ovarian cancer, NETs may be reshaped by the tumor microenvironment into factors that promote metastasis and immune evasion. In pregnancy-related disorders, NET abnormalities are more likely to reflect disruption of immune homeostasis at the maternal–fetal interface (34) (**Figure 5**).

**Figure 5:**
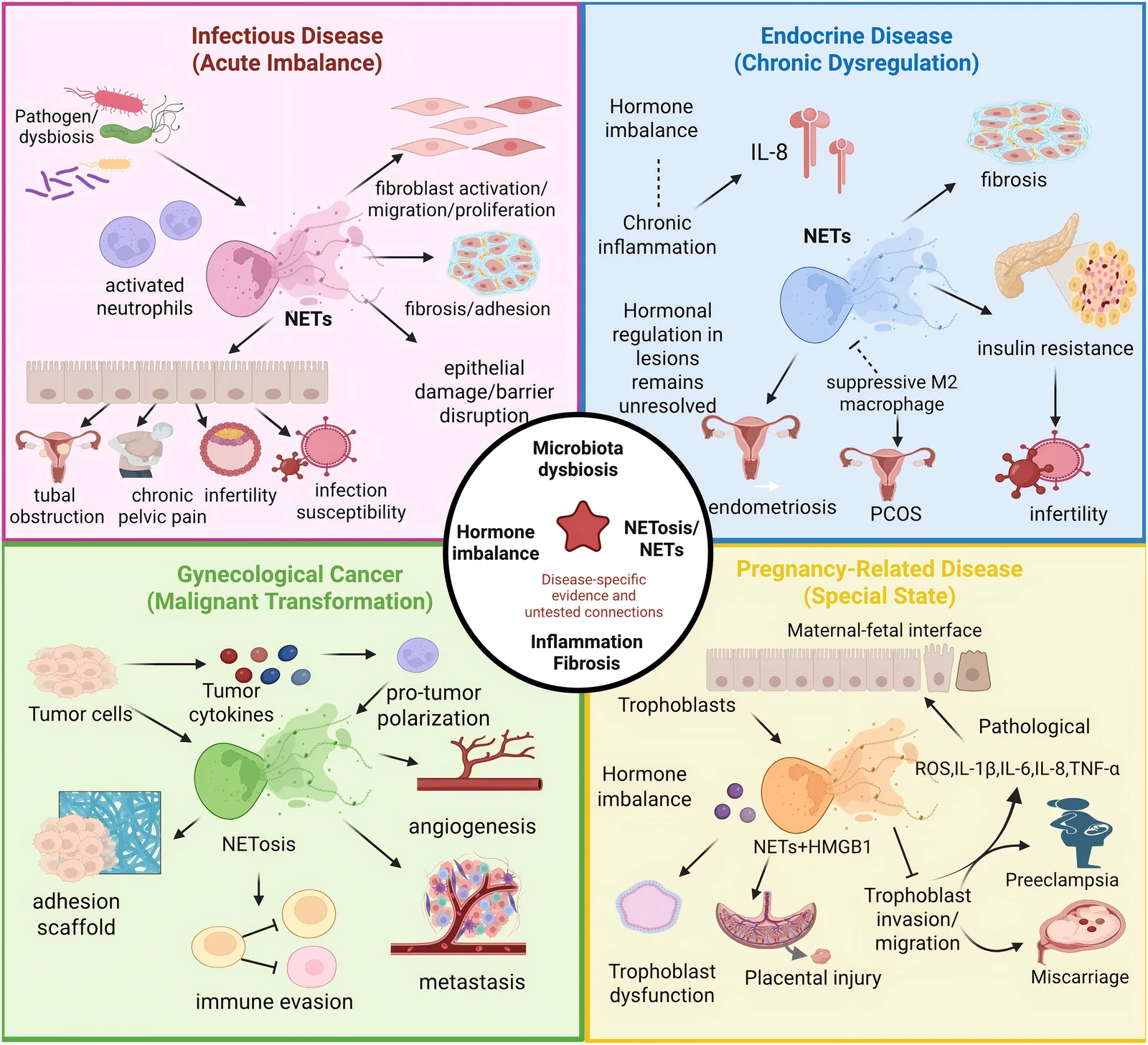
Disease-specific evidence contexts of hormone-, microbiota-, and NET-related alterations in gynecologic disorders. This figure summarizes disease-specific contexts in which hormonal alterations, microbiota changes, neutrophil extracellular trap (NET) formation, and inflammatory remodeling have been investigated across gynecologic disorders. Different diseases involve distinct dominant mechanisms. Infectious diseases provide relatively direct evidence linking microbial stimulation and NET activation. Chronic inflammatory disorders, endocrine-related conditions, malignancies, and pregnancy-associated complications show varying degrees of NET involvement, but the strength and directionality of causal relationships differ substantially among disease settings. The figure is intended to organize currently available evidence rather than imply that all gynecologic diseases share a common initiating mechanism. Disease-specific pathways should therefore be interpreted according to the available experimental and clinical evidence. Solid arrows indicate supported relationships within specific disease contexts, whereas dashed arrows indicate hypotheses or unresolved connections.

### 5.1 Infectious diseases: acute and mucosal disequilibrium, represented by pelvic inflammatory disease and bacterial vaginosis

Infectious diseases represent the setting in which the most direct links within the “hormone–microbiota–NET framework” can be established, as pathogens and their associated molecular patterns can directly induce neutrophil activation and NETosis. In this category, the strongest current evidence does not concern the “hormone–NET” or “GAGs–PAD4” pathways, but rather the rapid release of NETs driven by pathogens or microbiota imbalance, together with the dual role of NETs in host defense and tissue injury. Accordingly, PID may be more appropriately understood as a model of acute immune disequilibrium, in which NETs may both limit the spread of infection and, when excessively formed or insufficiently cleared, contribute to subsequent fibrosis and adhesions.

#### 5.1.1 Pelvic inflammatory disease: NET-mediated tissue injury and sequelae

Pelvic inflammatory disease and its sequelae represent a typical context in which NETs shift from defensive effectors to amplifiers of tissue injury. In adhesion tissues from SPID rats, autophagy and NET expression were found to be concomitantly increased, and *in vitro*, NETs promoted fibroblast proliferation (81). In a mouse model of experimental peritoneal adhesions and in human adhesion biopsies, NET deposition was observed at sites of injury, while DNASE1/DNASE1L3-related interventions significantly reduced peritoneal adhesions in mice (88). In a mouse model of genital tract *Chlamydia* infection, neutrophils were initially involved in host defense, but their sustained recruitment and prolonged survival were associated with subsequent tissue damage (97). Thus, the available evidence, derived mainly from animal models and *in vitro* studies, supports a role for NETs in pelvic adhesions and fibrosis; however, in patients, the causal relationship between NETs and long-term sequelae remains unresolved. Therefore, NET-associated tissue injury in PID has substantial biological plausibility and preliminary experimental support.

#### 5.1.2 Bacterial vaginosis: dysbiosis-associated extracellular trap formation and mucosal inflammation

Bacterial vaginosis (BV) provides a distinct mucosal context for the hormone–microbiota–NET framework because it is characterized by a shift from *Lactobacillus*-dominated communities toward polymicrobial dysbiosis rather than by infection with a single invasive pathogen. Recent human vaginal-sample studies provide direct evidence that extracellular traps are present in BV. Ramírez-Ledesma et al. detected LL-37-associated extracellular DNA traps in vaginal samples from women with BV and other infectious or inflammatory vaginal conditions; notably, extracellular traps were detected in all BV samples examined, although the cellular source could not be definitively assigned to neutrophils in that study. More recently, Prado-Sanhueza et al. directly characterized NETs and macrophage extracellular traps in vaginal smears from women with BV and normal microbiota. BV samples showed substantially higher NET counts and a significant increase in diffuse NETs, while *Gardnerella vaginalis*-positive samples showed enrichment of spread NETs (84, 85).

Complementary experimental evidence indicates that BV-associated bacterial communities can induce rapid and sustained neutrophil recruitment, accompanied by epithelial junction disruption and increased vaginal barrier permeability (86). These findings support a relationship among vaginal dysbiosis, neutrophil activation, extracellular trap formation, and mucosal inflammation. However, the available studies do not yet establish whether NETs are upstream drivers of epithelial injury, secondary products of local inflammation, or components of an antimicrobial response that becomes maladaptive when excessive or insufficiently cleared. BV should therefore be regarded as a clinically relevant model of microbiota–neutrophil/NET interaction, while the balance between protective microbial containment and NET-associated inflammatory injury remains to be defined.

### 5.2 Endocrine-related diseases: chronic dysregulation, represented by endometriosis and PCOS

Unlike infectious diseases, NET abnormalities in endocrine-related disorders are more likely to manifest as part of persistent low-grade inflammation, immune disequilibrium, and tissue remodeling, rather than as a single acute defensive response. In this category, neutrophil-associated inflammatory indices have been examined clinically in endometriosis, whereas a direct hormone–NET mechanism remains more theoretical and requires disease-specific validation (98).

#### 5.2.1 Endometriosis: NET-driven chronic inflammation and fibrosis

Among endocrine-related gynecologic disorders, endometriosis has been relatively more extensively studied in the context of NETs. Investigators compared circulating NET levels between surgically confirmed endometriosis patients and surgical controls without endometriosis, and they found elevated circulating NET levels in the endometriosis group (80). In a case–control study, peripheral blood levels of multiple NET-related markers, including cell-free DNA (cf-DNA), nucleosomes, and NE, were compared between patients with endometriosis and controls, and all were significantly increased in the patients (24). Case–control studies in patients therefore provide relatively direct clinical evidence that NET-related markers are elevated and associated with pain and infertility. However, it remains difficult to determine whether NETs are upstream drivers of lesion progression, amplifiers that promote fibrosis and immune escape, or merely by-products of the chronic inflammatory environment. In addition, key pathways such as estrogen-enhanced NETosis and microbiota-mediated modulation of the NET threshold through GAGs–PAD4 have only been indirectly supported in local endometriotic lesions (8, 80).

#### 5.2.2 Polycystic ovary syndrome: preliminary evidence for NET involvement in metabolic and inflammatory regulation

Researchers measured NET markers in the serum and follicular fluid of patients with PCOS and found both to be elevated relative to controls. In DHEAS-induced PCOS rats, degradation of NETs with DNase I improved glucose metabolism; further *in vitro* hepatocyte experiments showed that NET-conditioned medium reduced insulin sensitivity, glucose uptake, and glucose utilization, while downregulating glycolysis-related molecules (90). Transfer of fecal microbiota from PCOS patients to recipient mice, or colonization with *Bacteroides vulgatus*, induced ovarian dysfunction and insulin resistance, altered bile acid metabolism, and reduced IL-22 levels (89). In high-fat-diet mice, increased ET release and reduced clearance were observed, and blockade of TLR7/9 or inhibition of ET formation improved metabolic inflammation and glucose homeostasis (99). Other investigators found that NE treatment could directly induce hepatocellular insulin resistance and that NE-deficient mice exhibited better glucose tolerance and insulin sensitivity under a high-fat diet (100). Taken together, these findings suggest an association among elevated NETs, microbiota abnormalities, and metabolic dysfunction in PCOS, while animal and *in vitro* studies further indicate that NETs may participate in the development of insulin resistance. Nevertheless, most of this evidence comes from small-sample studies, exploratory observations, and experimental systems.

### 5.3 Gynecologic malignancies: malignant transformation, with ovarian cancer as the best-characterized NET model

In the tumor context, the significance of NETs differs from that in both infection and chronic inflammatory disorders. Here, NETs are no longer primarily manifestations of host defense, but may instead be co-opted by the tumor microenvironment as tools that promote metastasis, angiogenesis, and immune escape. Ovarian cancer is a key focus not only because NET-related studies are relatively abundant but also because the biological process of peritoneal dissemination offers a comparatively clear model for examining the relationship between NETs and tumor microenvironment remodeling (43). More broadly, tumor-associated neutrophils can show context-dependent anti- or pro-tumor polarization, but such evidence should be treated as indirect background rather than ovarian cancer-specific NET evidence (101).

#### 5.3.1 Ovarian cancer: NET-driven metastasis and immune escape

Ovarian cancer is currently the gynecologic malignancy in which NETs have been most extensively studied (40–42). In orthotopic ovarian cancer models, early ovarian tumors were shown to induce neutrophil infiltration into the omentum, where NETs could bind to and trap ovarian cancer cells, thereby promoting omental colonization. Omental colonization was markedly reduced in mice with neutrophil-specific PAD4 deficiency or after pharmacological inhibition of PAD4-mediated NET formation (43). NETs were also found to induce CXCL13 expression in the omentum, recruiting innate-like B cells into the premetastatic omentum and ultimately enhancing omental metastasis (102). Tumor-induced NETosis may further allow NETs to envelop tumor cells, thereby preventing direct contact between tumor cells and CD8+ T cells or NK cells; inhibition of PAD4/NETosis enhanced synergy with immune checkpoint therapy (103). These studies support a role for NETs in peritoneal implantation, immune escape, and the remodeling of the tumor microenvironment, together providing strong mechanistic clues for the pro-tumorigenic role of NETs (104). However, whether hormonal status modulates NET function in ovarian cancer and whether NET-targeted inhibition can yield genuine clinical benefit remain preclinical questions. NETs should therefore be regarded as important participants in the ovarian cancer pathological microenvironment.

#### 5.3.2 Cervical cancer: emerging evidence for NET-mediated tumor progression

Earlier evidence linking neutrophil-dominant inflammation to cervical cancer was largely indirect, including associations between an elevated neutrophil-to-lymphocyte ratio and unfavorable clinical outcomes. More recent studies, however, provide direct evidence for NET involvement in cervical cancer progression. Ning et al. demonstrated increased neutrophil infiltration and NET formation in cervical cancers with lymph node metastasis. Mechanistically, cervical cancer-derived S100A7 promoted neutrophil chemotaxis and ROS-dependent NET formation, whereas NETs enhanced cervical cancer cell migration through TLR2-associated p38-MAPK/ERK/NF-κB signaling and promoted lymphangiogenesis and lymphatic dissemination. DNase I-mediated NET degradation reduced lymph node metastasis in experimental models (91).

More recently, Wang et al. reported elevated NET-related signals in cervical cancer tissues and sera, particularly in HPV-positive disease. Their clinical and experimental analyses implicated an HPV-associated CXCL8–CXCR1/2–ROS–p21 pathway in NET induction, while NETs promoted cervical cancer cell proliferation, angiogenesis, invasion, and migration. High tissue CitH3 was associated with aggressive clinicopathological features, and experimental NET degradation attenuated tumor-promoting effects (92). These findings substantially strengthen the biological plausibility of NET-mediated tumor progression in cervical cancer. Nevertheless, the direct mechanistic evidence remains concentrated in a limited number of studies, and independent prospective validation is required before NET-related biomarkers or NET-targeted interventions can be considered clinically actionable.

#### 5.3.3 Endometrial cancer: tissue NETosis and circulating NET-related biomarkers

Direct patient-based evidence indicates that NETosis occurs in endometrial cancer tissue and is accompanied by circulating NET-related abnormalities. Ronchetti et al. examined endometrial cancer specimens using immunohistochemistry and confocal immunofluorescence for citrullinated histone H3 (CitH3), NE, histone H2B, and DNA. NETosis-associated features were detected across tumor grades, including extracellular colocalization of DNA, NE, and CitH3, whereas healthy endometrial controls lacked comparable CitH3 staining. The same study also reported associations among circulating cf-DNA, CitH3, and systemic inflammatory features (93).

In a subsequent clinical study involving 98 patients with endometrial cancer and 45 healthy women, Seo et al. found significantly elevated circulating histone–DNA complexes, cell-free double-stranded DNA, and NE in patients with endometrial cancer. Higher cell-free dsDNA was independently associated with poorer progression-free and overall survival after adjustment for clinicopathological variables (94). These observations support the presence of NET-associated inflammation in endometrial cancer and suggest potential biomarker relevance. However, circulating cell-free DNA is not specific to NETosis, and the current studies do not establish whether NETs are causal drivers of tumor progression or secondary consequences of tumor-associated inflammation. Likewise, despite the well-established endocrine dependence of many endometrial cancers, a direct estrogen–PAD4–NET causal pathway has not yet been demonstrated in endometrial cancer tissue.

### 5.4 Pregnancy-related diseases: a special physiological state, represented by preeclampsia

Research on NETs in pregnancy-related disorders is distinctive because pregnancy itself is a physiological condition characterized by the simultaneous remodeling of hormones, immunity, and the local microenvironment. Normal pregnancy requires a balance between antimicrobial defense and maternal–fetal tolerance; thus, NETs in this setting may have physiological functions but may also become pathological when regulation is disrupted. Preeclampsia is an appropriate representative disease because it involves maternal inflammation, placental dysfunction, and alterations in the hormonal milieu, although most current evidence remains limited to cross-sectional observations and *in vitro* mechanistic studies (105, 106).

Recent studies focused on implantation and maternal–fetal interface biology provide useful context for this pregnancy-related branch of the framework. Lund et al. (39) summarized coordination among endometrial microbiota, cytokines, and growth factors at the maternal–fetal interface, whereas Lund et al. (74) used single-cell RNA sequencing to show that LPS can disrupt cell communication and inflammatory signaling at that interface. These studies are relevant to the microbiota/microbial product–immune environment part of the framework, but they concern implantation-related pathology and experimental LPS exposure rather than demonstrating a reproductive tract dysbiosis–NET mechanism in preeclampsia.

#### 5.4.1 Preeclampsia: NET-mediated placental dysfunction

NET-related abnormalities in preeclampsia and their association with trophoblast injury have received some support from patient studies and *in vitro* evidence (96, 105). However, it remains unclear whether NET-related changes occur before placental dysfunction or represent a secondary amplification response to injury, whether the progesterone-mediated “brake” on NETs truly fails in preeclampsia, and whether microbiota abnormalities contribute to this process. NETs may therefore participate in inflammatory amplification and placental dysfunction in preeclampsia, but their causal position in disease progression still requires clarification through longitudinal and interventional studies (95).

## 6 Controversies and knowledge gaps

Although the hormone–microbiota–NET framework offers an integrative perspective for understanding interactions among immunity, endocrine regulation, and microecology in gynecologic diseases, this framework remains at the stage of hypothesis integration and evidence accumulation rather than a fully validated unified model. Its main challenge is not whether local supportive evidence exists, but rather that the sources, strength, and generalizability of the evidence across its different pathways are uneven. Accordingly, the more pressing question is not simply whether this framework exists as a useful heuristic, but which components are already supported by relatively direct gynecologic evidence, which links still rely mainly on extrapolation from other systems, and which diseases truly share common mechanisms rather than only superficial similarities. Clarifying these controversies and knowledge gaps is essential for avoiding overinterpretation and for guiding the field toward testable research pathways.

### 6.1 Asymmetry in evidence sources: a persistent gap between direct gynecologic evidence and cross-system extrapolation

There is marked asymmetry in the evidence supporting the four core pathways of the hormone–microbiota–NET framework. First, although studies on the hormonal regulation of NETs are relatively numerous, most derive from non-gynecologic *in vitro* systems and animal models. For example, the molecular mechanisms by which estrogenic signaling may promote NETosis and progesterone may restrain NET release have been established largely in neutrophil models, pregnancy immunology, or other inflammatory settings, whereas direct validation in authentic gynecologic patient samples remains limited (26, 35). Second, direct induction of NETs by pathogens or microbial products through classical inflammatory pathways such as PAMP–TLR signaling is supported by relatively direct evidence in infectious diseases, but such evidence is concentrated mainly in acute infection or vaginitis settings and cannot be naturally extrapolated to chronic disorders such as endometriosis or PCOS. Third, although the hypothesis-generating GAG–PAD4 mechanism is one of the most innovative links in this framework, its core evidence currently derives mainly from *in vitro* biochemical studies, and its existence, regulatory strength, and pathological significance in local gynecologic microenvironments remain to be verified. Fourth, the notion that NETs may, in turn, influence the hormonal microenvironment is theoretically attractive, but at present, it is supported largely by correlative observations, *in vitro* experiments, or indirect evidence from other systems, and it remains far from direct causal validation in gynecologic diseases.

### 6.2 Correlation does not equal causation: many mechanistic pathways remain unclosed

At present, many studies on NETs in gynecology remain limited to observations of elevated biomarkers or NET deposition within tissues. Such evidence may indicate association, but it is insufficient to determine whether NETs are disease drivers, inflammatory amplifiers, or merely by-products of tissue injury. In endometriosis, PCOS, and preeclampsia, for example, studies have reported increased NET-related markers and associations with pain, insulin resistance, or placental injury, yet most are cross-sectional and cannot determine whether NET elevation occurs before, during, or after disease development (107). Altered microbiota and increased NETs often occur concurrently, but the directional relationship between them remains unclear: microbiota dysbiosis may drive NETosis, while NET-associated proteases and inflammatory conditions may, in turn, disrupt mucosal or epithelial homeostasis and shape an abnormal microenvironment (19, 86). Thus, moving from co-occurrence to causation will require more longitudinal sampling, interventional experiments, and multilevel models (108, 109).

### 6.3 A “common framework” does not necessarily imply a “common mechanism”: different diseases may not share the same pathological logic

The integrative value of the “hormone–microbiota–NET framework” lies in its ability to provide a cross-disease explanatory framework, but this does not mean that all gynecologic diseases operate through the same mechanism. In infectious diseases, NETs are more likely to reflect acute responses to pathogens, with the central tension lying between host defense and tissue injury. In chronic disorders such as endometriosis or PCOS, by contrast, NETs may constitute part of persistent low-grade inflammation and tissue remodeling, with different temporal dynamics and microenvironmental contexts. In ovarian cancer, NETs may be co-opted by the tumor microenvironment to promote metastasis and immune escape. Thus, even when “NET elevation” is observed across diseases, its biological meaning may differ fundamentally between contexts.

### 6.4 In chronic inflammatory settings, the microbiota–NET connection remains substantially weaker than in infection

Within this framework, the microbiota–NET connection is most readily established in infectious contexts. Pathogen-associated molecular patterns can activate neutrophils and induce NETosis, and this pathway has a relatively direct biological basis with some support from gynecologic evidence. However, in chronic non-infectious disorders, there is still no equally strong evidence explaining how microbiota alterations persistently influence the threshold for NET formation. In diseases such as endometriosis, PCOS, and preeclampsia, microbiota dysbiosis, chronic inflammation, and NET abnormalities are often observed together, but it remains difficult to determine which component is the initiating disturbance and which primarily drives pathological amplification.

### 6.5 The hypothesis-generating GAG–PAD4 mechanism: high priority but weakly validated

At present, the central finding in this pathway is that GAGs can enhance the calcium sensitivity of PAD4 *in vitro*, thereby offering a possible explanation for the “calcium paradox.” However, extrapolating this *in vitro* phenomenon to real pathological processes in gynecologic mucosal microenvironments still involves multiple unverified steps: whether local GAG profiles truly change in response to microbiota alterations, whether such changes are sufficient to affect intracellular PAD4 activity in neutrophils, whether the effect is strong enough to alter the threshold for NETosis, and whether these changes correlate with disease phenotypes in patient samples (87).

### 6.6 Whether hormonal regulation of NETs has gynecologic specificity still requires further validation

The opposing regulatory effects of estrogen and progesterone on NETs are among the key pillars of this framework. However, it remains uncertain whether this regulation is consistent across different gynecologic diseases, tissue sites, and hormonal settings. First, circulating hormone levels are not equivalent to local tissue exposure, especially in endometriotic lesions, tumor microenvironments, and the maternal–fetal interface, where local steroid metabolism may differ substantially from peripheral status. Second, menstrual-cycle stage, hormonal contraception, menopausal status, pregnancy, and obesity/metabolic dysfunction may all alter the direction and magnitude of hormone–immune interactions. Third, neutrophils themselves display substantial functional plasticity, and their responses to the same hormonal stimulus may vary depending on their activation state.

### 6.7 Non-uniform sample sources and NET detection methods limit cross-study comparability

Another important but often underestimated problem is the methodological heterogeneity of NET research. Different studies use peripheral blood, vaginal secretions, ascites, lesion tissue, placental tissue, and follicular fluid, among other sample sources, yet these specimens reflect different biological levels and are not necessarily directly comparable. In addition, NET detection methods are not standardized: some studies use cell-free DNA, whereas others assess MPO–DNA complexes, CitH3, NE, or tissue immunostaining, and these markers do not necessarily represent the same biological process. Some are more reflective of general cell damage or inflammatory burden, whereas others are more specific to NETosis itself. Without standardized sampling strategies and combined biomarker systems, it is difficult to establish a truly comparable “axis signature,” which is one of the most practical current barriers to moving from mechanistic discussion toward clinical translation (108–110).

### 6.8 NET as a double-edged sword: antimicrobial defense versus inflammatory injury

NETs should not be regarded uniformly as pathological structures. Their original biological function is antimicrobial containment, and this function remains particularly relevant at mucosal surfaces exposed continuously to microorganisms. NETs can trap and kill bacteria, while *C. albicans* can induce NET formation and is susceptible to NET-mediated killing in both yeast and hyphal forms (45, 111). PAD4-dependent NET formation has also been shown to contribute to antibacterial defense: Pad4-deficient neutrophils exhibit defective NET formation and NET-mediated bacterial killing, and Pad4-deficient mice show increased susceptibility in an experimental necrotizing-fasciitis model (49).

This protective function has direct relevance to the female reproductive tract. In a murine model of vulvovaginal candidiasis, Yano and Fidel found that vaginal conditions supporting NET formation were associated with effective neutrophil antifungal activity and fungal clearance, whereas impaired NET-forming capacity was associated with persistent fungal burden and susceptibility to chronic vaginitis (70). Nevertheless, the contribution of NETs to microbial defense is not uniform across all infections. For example, PAD4 deficiency did not increase bacteremia in a polymicrobial sepsis model and partially ameliorated endotoxemic shock, indicating that the balance between antimicrobial benefit and inflammatory injury is highly context-dependent (112).

Conversely, excessive, persistent, or inadequately cleared NETs can expose histones and proteases, amplify inflammatory signaling, injure epithelial and endothelial barriers, and promote fibrosis, thrombosis, or tumor-supportive inflammation. Thus, the relevant biological question is not whether NETs are globally protective or pathogenic, but under which conditions their antimicrobial benefit is outweighed by collateral tissue injury. In gynecologic disease, this balance is likely to vary according to active infection status, pathogen burden, anatomical compartment, pregnancy state, timing of the response, and NET clearance capacity.

This duality constitutes an important safety boundary for therapeutic translation. Global PAD4 inhibition or indiscriminate NET degradation during active genital tract infection could theoretically impair antimicrobial containment, whereas targeting persistent NETs after pathogen control or in predominantly sterile inflammatory or malignant settings may have a different risk–benefit profile. Future intervention studies should therefore incorporate microbiological surveillance, infection-related safety endpoints, and disease stage-specific patient selection rather than assuming that suppression of NET formation is universally beneficial.

### 6.9 The most important future research priorities

In light of the above controversies, future studies should shift from asking whether this framework is conceptually appealing to testing which of its individual links genuinely hold true. At least four priorities deserve particular attention. First, standardized NET detection systems should be established according to disease type, sample source, and cycle/hormonal status. Second, longitudinal cohorts and interventional studies are needed to define the temporal sequence among microbiota dysbiosis, hormonal fluctuations, and NET changes. Third, the relationships among local GAG profiles, PAD4 activity, and the threshold for NET formation should be directly validated in gynecologic tissues and mucosal samples. Fourth, it is essential to determine whether reproducible framework-related phenotypes exist across different diseases so that highly heterogeneous patients are not forced into a single explanatory model. Only after these fundamental questions are progressively resolved can the “hormone–microbiota–NET framework” evolve from an intellectually stimulating integrative hypothesis into a research framework with real explanatory and translational values.

## 7 Clinical translation: from concept to validation—current barriers and future directions

The hormone–microbiota–NET framework provides a testable integrative approach for understanding shared pathogenic mechanisms in gynecologic diseases. However, the principal bottleneck in clinical translation lies not in the lack of candidate targets but in the absence of standardized detection methods, patient stratification strategies, and clearly defined endpoints. This section focuses on these core challenges, systematically outlining the major barriers faced by framework-informed diagnostic and therapeutic strategies during clinical translation and, on that basis, proposes directions for future research (10, 24, 90, 113).

### 7.1 NET-related biomarkers and candidate combined profiles: evidence boundaries

NET-related readouts should be interpreted according to what is measured rather than treated as interchangeable estimates of NETosis. cf-DNA and soluble NE provide non-specific evidence of extracellular DNA burden and neutrophil activation, respectively, whereas MPO–DNA complexes and CitH3-based measurements require assay-specific interpretation and complementary assessment (110, 114). Representative gynecologic studies report circulating-marker associations and local tissue findings, but these observations do not establish a clinically validated, cross-disease hormone–microbiota–NET diagnostic panel (93, 94, 115). **Table 4** distinguishes readout type, specificity, complementary methods, confounders, and evidence in the studies reviewed. Analytical performance, disease association, and clinical utility should be evaluated separately. Any combined profile requires validation in its intended specimen and population, including prospective assessment of its added value beyond established clinical predictors.

**Table 4:** NET-related readouts: specificity, complementary assessment, and evidence boundaries in gynecologic research.

Marker/method	Readout type and specimen	Specificity for NET formation/interpretive limit	Recommended complementary assessment	Major confounders	Evidence in gynecologic research and validation status*
cf-DNA	Soluble extracellular DNA; serum/plasma and local fluids.	Low specificity. Reflects extracellular DNA burden and cannot identify a neutrophil source or establish NET formation alone.	A validated NET-associated protein–DNA assay and, where feasible, structural confirmation.	Non-NET cell death; serum/plasma differences; delayed processing; storage and freeze–thaw conditions.	Exploratory human associations, including endometrial cancer. The reviewed studies do not establish a stand-alone NET-specific diagnostic test (93, 94).
MPO–DNA complexes	Soluble protein–DNA complexes; plasma or other explicitly validated sample matrices.	NET-associated, but not independently confirmatory. Plasma assays can yield non-specific signals; performance is assay- and matrix-dependent.	Independent structural assessment and/or a separately validated CitH3-based assay; appropriate assay controls.	Non-specific antibody binding; matrix interference; antibody selection; calibration and threshold differences.	Research application is reported in PCOS-related work; this does not establish clinical diagnostic validity. Validation should match the exact assay, specimen, and disease (90; methodological caution: 110).
CitH3-based measurements	Soluble antigen or nucleosome-associated signal in fluids; structural staining in tissues/smears. These formats are not interchangeable.	Supports histone citrullination, not proof of extracellular NET structures by itself. Absence does not exclude all NET-forming pathways.	Extracellular DNA–MPO/NE colocalization; explicit identification of the CitH3 assay format.	Antibody specificity and lot; calibrator composition; fixation and background staining; assay format.	Human tissue and circulating-marker studies exist in endometrial and ovarian cancer. These represent exploratory disease evidence, not a universally validated gynecologic NET test (93, 115).
Soluble NE	Soluble protein; serum/plasma or local fluids.	Low specificity for NET formation. Cannot distinguish soluble enzyme release from NET-bound NE.	Extracellular DNA–NE colocalization and a complementary NET-associated biochemical readout.	Inflammatory burden; neutrophil abundance/activation; specimen type and pre-analytical handling.	Human association studies include endometrial cancer; supportive inflammatory readout rather than a stand-alone NET assay (94).
Immunofluorescence colocalization	Structural readout in tissue, smears, or local samples: extracellular DNA with MPO/NE; CitH3 as an additional stain where appropriate.	Relatively direct evidence of extracellular NET structures when morphology and colocalization are adequately controlled; a fixed image does not establish release dynamics.	A matched soluble-marker assay or functional assessment, where feasible.	Antibody/background staining; fixation; image acquisition; field selection; segmentation and threshold settings.	Local human observations include endometrial cancer and vaginitis. Morphological evidence should not be equated with validated diagnostic thresholds (71, 93).
Live-cell imaging/functional induction assays	Dynamic or functional readout in isolated neutrophils, depending on the method.	Can examine release kinetics or stimulus responsiveness. *Ex vivo* inducibility does not quantify the *in vivo* NET burden.	Cell-viability assessment, extracellular morphology, and an independent NET-associated readout.	Isolation-related activation; cell purity; stimulus, dose, timing, and culture conditions.	Functional hormone-response work is represented by pregnancy-associated studies; this is mechanistic support, not a routine gynecologic diagnostic test (35).
PAD4 expression/activity assays	Upstream molecular or enzymatic readout in cells, tissues, or biochemical systems; neither a soluble NET-remnant assay nor structural proof of NETs.	PAD4 abundance, enzymatic activity, and completed NET release are distinct measurements. Expression or activity alone does not establish NET formation.	Direct NET readouts plus appropriately controlled perturbation when testing causality.	Cellular composition; calcium conditions; substrate and matrix effects; expression–activity discordance.	Mechanistic evidence includes ovarian cancer models; GAG-dependent PAD4 modulation remains biochemical/hypothesis-level in this framework (31, 43).

Soluble-marker levels and structural observations should not be treated as interchangeable measures of NETosis. CitH3 staining is complementary rather than mandatory for every NET-forming pathway. Suggested marker combinations require specimen- and assay-specific validation; agreement between imperfect markers does not by itself establish diagnostic specificity. Serum and plasma results should not be pooled without justification. Record anatomical site, infection status, hormonal treatment, pregnancy status, and cycle phase where relevant. Methodological basis: Matta et al. (108)Hayden et al. (110)Thålin et al. (114), and Guiducci et al. (50). These studies inform assay interpretation but do not constitute gynecologic clinical validation. Specimens shown indicate research contexts, not a claim that every assay is validated in every listed matrix.

cf-DNA, cell-free DNA; CitH3, citrullinated histone H3; GAGs, glycosaminoglycans; MPO, myeloperoxidase; NE, neutrophil elastase; NETs, neutrophil extracellular traps; PAD4, peptidylarginine deiminase 4; PCOS, polycystic ovary syndrome.

*Validation status describes the representative studies considered in this narrative review, not a formal evidence-grading exercise. Analytical assay validation, association with disease, and demonstrated clinical utility are distinct. None of the entries should be interpreted as establishing a universally applicable hormone–microbiota–NET diagnostic panel.

### 7.2 Five core translational barriers

Barrier 1: Severe lack of standardization in NET detection

Current situation: Detection methods are highly heterogeneous, with substantial variation in sample type (serum vs. plasma), processing procedures, antibody sources, and threshold definitions (114).

What needs to be addressed: Technical standards for NET detection should be established, with clear specifications for pre-analytical handling, analytical methods, and quality-control criteria (110, 116–119).

Barrier 2: Results from different sample sources are not directly comparable

Current situation: Baseline levels differ between serum and plasma, and coagulation may lead to artifactual elevation. Delayed processing, repeated freeze–thaw cycles, and storage duration can also affect results. In addition, the impact of sampling during menstruation versus outside the menstrual period remains unclear.

What needs to be addressed: The optimal sample type and collection window for gynecologic NETs research should be defined, and standardized operating procedures for sample handling should be established.

Barrier 3: Physiological and pathological NETs are difficult to distinguish

Current situation: Pregnancy-associated studies indicate that NET-related activity can occur outside overt disease; however, the evidence reviewed here does not establish menstrual cycle-specific reference intervals for the non-pregnant reproductive tract. Physiological and pathological findings therefore require interpretation within the relevant sampling and clinical context (35).

Barrier 4: Complete lack of criteria for patient stratification

Current situation: Framework-related status is markedly heterogeneous across patients and across disease stages, such as estrogen-dominant versus atypical endometriosis, or obese versus lean PCOS. However, most current studies still use case–control designs and treat each disease as a homogeneous entity (120–122).

What needs to be addressed: Large-scale cohort studies should be conducted to identify clinically meaningful subtypes based on integrated hormonal, microbiota, and NET profiles.

Barrier 5: No consensus on the selection of interventional endpoints

Current situation: Candidate endpoints include symptom improvement (e.g., pain scores), clinical events (e.g., recurrence rate and pregnancy rate), and changes in framework-related biomarkers (e.g., restoration of hormone ratios and reduction in NETs) (123–125).

What needs to be addressed: Disease-appropriate clinical endpoints should be prespecified. NET-related biomarker changes may be included as exploratory mechanistic outcomes, but should not be treated as surrogate measures of clinical benefit without validation for the intended context.

### 7.3 Potential interventional targets: a staged validation pathway

Potential strategies involving hormonal modulation, microbiota-directed approaches, and NET/PAD4 targeting should first be evaluated as distinct interventions. The use of a treatment for an established indication does not demonstrate that its benefit is mediated through the proposed hormone–microbiota–NET framework. Likewise, experimental effects in disease models do not, by themselves, establish clinical efficacy or safety. Combination approaches should therefore initially be examined in relevant preclinical systems, with comparison against the individual components, rather than proposed as immediately actionable clinical regimens.

**Table 5** organizes the proposed research pathway into preclinical and analytical validation, translational human research, early clinical feasibility, and therapeutic efficacy trials. Advancement to interventional studies should depend on candidate- and indication-specific evidence, including reliable measurements, a justified target population, and an adequate safety rationale. In view of the antimicrobial functions discussed in Section 6.8, assessment should address active infection, microbial burden, preservation of host defense, and pregnancy-related risks where relevant. Clinical benefit should be evaluated using disease-appropriate outcomes; reductions in NET-related markers alone should not be interpreted as therapeutic success or as validated surrogate endpoints.

**Table 5:** Proposed staged pathway for validating hormone–microbiota–NET-informed biomarkers and interventions.

Stage	Research objective	Suitable study design	Principal readouts	Conditions for progression
1. Preclinical and analytical validation	Define reliable NET readouts; test disease-specific mechanisms, including the GAG–PAD4 hypothesis; assess individual interventions before combinations.	Assay development studies; relevant cell/*ex vivo* and animal models; controlled genetic or pharmacological perturbation; preclinical comparisons of individual and combined approaches.	Assay specificity and reproducibility; structural and functional NET changes; PAD4 activity where relevant; tissue injury, microbial control, and safety signals.	Demonstrate fit-for-purpose measurements and reproducible, context-specific effects. Candidate interventions require an adequate efficacy–safety rationale before human exposure.
2. Translational human research	Determine whether local findings and combined profiles are reproducible in defined diseases; examine temporal ordering and clinical associations.	Observational cohorts with longitudinal sampling; paired local/systemic samples where feasible; prespecified hormonal, microbiota, and NET assessments; independent validation cohorts.	Temporal associations; local/systemic concordance; disease-specific risk or prognostic performance; added information beyond established clinical predictors.	Confirm assay performance in the intended specimen and population; reproduce associations or predictive performance independently. Observational associations do not alone justify treatment.
3. Early clinical feasibility—conditional	For a sufficiently supported candidate and indication, assess tolerability, feasibility, and evidence of target modulation; do not infer efficacy from biomarker change alone.	Small, closely monitored early-phase studies only after candidate-specific preclinical evaluation and appropriate ethical/regulatory review.	Safety and tolerability; infection-related events; feasible sampling; candidate-specific exposure and target-engagement measures where applicable.	Require an acceptable indication-specific safety profile, feasible procedures, and interpretable biological readouts. Pregnancy-related use requires a separate safety rationale.
4. Therapeutic efficacy trials—conditional	Test whether a selected intervention produces clinically meaningful benefit. Combination regimens require evidence supporting their individual components and added value.	Disease-specific controlled trials with prespecified populations, comparators, and clinical endpoints, following adequate earlier-stage evidence.	Clinical outcomes appropriate to the disease, such as symptoms, recurrence, or reproductive outcomes; adverse events. NET-related markers remain secondary/exploratory unless validated for the intended role.	Clinical adoption would require demonstrated benefit–risk and reproducibility. A decrease in NET-related markers is not sufficient evidence of therapeutic benefit.

This table is an author-proposed research pathway, not a statement that any candidate intervention is ready for clinical testing. Analytical validation and observational human research may proceed in parallel; the sequence is not intended to require proof of causality before exploratory biomarker studies. Progression to interventional stages depends on the specific candidate, indication, and safety evidence. The established use of a treatment for another indication does not validate the proposed combined framework or demonstrate a NET-mediated mechanism. The original directions are retained within the stages: detection standardization and GAG–PAD4 testing in Stage 1; combined biomarkers, hormone-related variability, and microbiota–NET directionality in Stage 2; conditional intervention development in Stages 3–4. Disease-specific stratification and preservation of antimicrobial defense are cross-cutting requirements.

NET, neutrophil extracellular trap; GAG, glycosaminoglycan; PAD4, peptidylarginine deiminase 4.

## 8 Conclusion and outlook

The hormone–microbiota–NET framework proposed in this review is intended as a testable heuristic framework for understanding shared pathogenic mechanisms across gynecologic diseases. The central premise is that gynecologic disorders—whether infectious diseases, endocrine disturbances, gynecologic malignancies, or pregnancy-related complications—may be more coherently interpreted through multidirectional interactions among hormones, microbiota, and NETs, provided that supported component-level observations are clearly separated from untested causal bridges.

### 8.1 Organizational value of the framework and its current limitations

This framework attempts to bridge several disciplines that have traditionally been studied in relative isolation—including reproductive endocrinology, microbiology, immunology, and oncology—and thus offers a shared language for interdisciplinary research in gynecologic diseases. It also suggests that distinct disease categories may be linked by certain underlying mechanisms. From acute infection, such as pelvic inflammatory disease, to chronic inflammatory conditions, such as endometriosis, and further to malignant transformation, as exemplified by ovarian cancer, NETs may represent a recurring mechanistic thread. This perspective may help overcome research boundaries imposed by disease-specific classification. In addition, the framework provides a new conceptual direction for clinical investigation, including combined assessment based on framework-related signatures and the idea of multimodal intervention targeting hormones, microbiota, and NETs, thereby reflecting an exploratory shift from symptom management toward etiologic intervention (37, 38, 126, 127). However, these organizational advantages remain largely conceptual at present. For this framework to truly advance the field, the priority is to identify the hypotheses most worthy of direct testing and the research directions most deserving of early investment.

### 8.2 The GAG–PAD4 mechanism as one falsifiable research hypothesis

The GAG–PAD4 mechanism is one hypothesis-generating component of this framework, rather than its established molecular center. Biochemical evidence justifies testing whether relevant GAG species influence PAD4 under gynecologic conditions, but does not establish that microbiota-dependent GAG changes regulate intracellular PAD4 or NET release *in vivo*. Testing should distinguish three questions: whether microbial status relates to local GAG composition, whether the relevant GAG species alter PAD4 activity in the appropriate compartment, and whether PAD4-dependent changes contribute to NET formation and disease-related outcomes. Negative results at any step would limit this proposed route without invalidating separately supported hormonal, microbial, or NET-related observations.

### 8.3 Research directions that should be prioritized

Based on the above hierarchy of hypotheses, the following research directions merit priority consideration.

Direction 1: Test the compartment-specific relevance of the GAG–PAD4 hypothesis

Core question: Do the relevant local GAG species affect PAD4 activity and NET formation in the disease compartment under study?

Suggested research strategy: Evaluate the proposed links separately, beginning with characterization of local GAG species and controlled biochemical or cellular tests. Distinguish extracellular PAD4 activity from intracellular PAD4-dependent NET formation, and include appropriate controls for PAD4-independent effects. Tissue and secretion sampling should match the intended disease compartment; vaginal measurements should not be assumed to represent endometriotic lesions, ovarian tumors, or the placenta. Subsequent disease-model studies can test whether manipulating the candidate link changes both the mechanistic readouts and a relevant phenotype. Negative findings should be used to revise or reject the proposed connection (128–130).

Direction 2: Prioritize analytical validation and harmonization of NET-related measurements

Core question: How can NET-related measurements be made analytically reliable, biologically interpretable, and comparable across laboratories?

Suggested research strategy: Method-development studies should prioritize analyte definition, specimen-specific pre-analytical procedures, antibody specificity, suitable calibration materials, precision, recovery, dilutional behavior, and inter-laboratory reproducibility (110, 114). Soluble measurements should be evaluated alongside independent structural or functional NET assessments in representative samples (108). Automation may subsequently be investigated to improve throughput and workflow consistency, but it should not be assumed to improve NET specificity or replace analytical and clinical validation. Well-characterized cohorts should be used to investigate physiological variation and candidate reference distributions using validated assays before clinical decision thresholds are proposed.

Technical challenges: Major challenges include specimen-dependent interference, heterogeneity in analytes and assay formats, harmonization across laboratories, and the distinction between physiological variation, disease association, and clinical utility.

Direction 3: Conduct prospective cohort studies based on framework-related signatures

Core question: Do framework-related signatures, defined by hormones plus microbiota plus NETs, have independent clinical predictive value?

Suggested research strategy: Establish multicenter prospective cohorts including patients with representative diseases such as endometriosis, PCOS, and preeclampsia, together with healthy controls. Baseline samples should be collected using standardized procedures, with simultaneous assessment of hormones (E2, P4, and E2/P4), microbiota features (16S rRNA sequencing and *Lactobacillus* abundance), and NET-related markers (CitH3, NE, and MPO–DNA). Clinical endpoints, including disease progression, treatment response, recurrence, and pregnancy outcomes, should then be longitudinally followed to evaluate the independent predictive value of these framework-related signatures. Unsupervised approaches such as clustering analysis may help identify disease subtypes based on framework characteristics and explore their association with clinical outcomes.

Technical challenges: Multicenter coordination is organizationally demanding; large sample sizes are needed to preserve statistical power under multiple-testing conditions; follow-up periods are long, especially for recurrence and pregnancy outcomes; and the costs of microbiota and NETs analyses remain a practical concern.

### 8.4 Closing remarks

The value of this framework lies in distinguishing supported component-level observations from untested connections across gynecologic diseases. Future work should combine disease-specific and compartment-resolved studies, reliable NET measurements, and prospective human research to determine which links are reproducible and clinically relevant. The GAG–PAD4 mechanism is one candidate for such testing, not a prerequisite for the validity of all hormonal, microbial, or NET-related observations. The framework should be revised as evidence supports, limits, or refutes its proposed connections.
